# Comparative transcriptomics provides a strategy for phylogenetic analysis and SSR marker development in *Chaenomeles*

**DOI:** 10.1038/s41598-021-95776-z

**Published:** 2021-08-12

**Authors:** Wenhao Shao, Shiqing Huang, Yongzhi Zhang, Jingmin Jiang, Hui Li

**Affiliations:** 1 Research Institute of Subtropical Forestry, Chinese Academy of Forestry, Hangzhou, 311400 China; 2 Longshan Forest Farm of Anji County, Huzhou, 313300 China; 3 Guangzhou Institute of Forestry and Landscape Architecture, Guangzhou, 510405 China

**Keywords:** Biotechnology, Genetics

## Abstract

The genus *Chaenomeles* has long been considered an important ornamental, herbal and cash crop and is widely cultivated in East Asia. Traditional studies of *Chaenomeles* mainly focus on evolutionary relationships at the phenotypic level. In this study, we conducted RNA-seq on 10 *Chaenomeles* germplasms supplemented with one outgroup species, *Docynia delavayi* (*D. delavayi*), on the Illumina HiSeq2500 platform. After de novo assemblies, we generated from 40,084 to 49,571 unigenes for each germplasm. After pairwise comparison of the orthologous sequences, 9,659 orthologues within the 11 germplasms were obtained, with 6,154 orthologous genes identified as single-copy genes. The phylogenetic tree was visualized to reveal evolutionary relationships for these 11 germplasms. GO and KEGG analyses were performed for these common single-copy genes to compare their functional similarities and differences. Selective pressure analysis based on 6,154 common single-copy genes revealed that 45 genes were under positive selection. Most of these genes are involved in building the plant disease defence system. A total of 292 genes containing simple sequence repeats (SSRs) were used to develop SSR markers and compare their functions in secondary metabolism pathways. Finally, 10 primers were chosen as SSR marker candidates for *Chaenomeles* germplasms by comprehensive standards. Our research provides a new methodology and reference for future related research in *Chaenomeles* and is also useful for improvement, breeding and selection projects in other related species.

## Introduction

*Chaenomeles*, a genus within the subfamily Maloideae (Rosaceae) comprising four diploid (2n = 34) species^1^, is widely planted in East Asia and has important ecological, ornamental and economic value. In China, *Chaenomeles* has a cultivation history of more than 3000 years. Approximately 700 years ago, people began to recognize *Chaenomeles*’ medicinal values: their effect on curing rheumatoid arthritis and hypopepsia^2^. Currently, pharmacologists find that their leaves and flowers contain substantial prunasin; their seeds have a large amount of amygdalin; and their fruits contain abundant chemical ingredients, such as oleanolic acid, malic acid, pectinic acid, Chinese wolfberry citric acid, tartaric acid, citric acid, tannin, flavonoids, saponin and proanthocyanidins, which act as antioxidant and anticancer agents^3^. The remaining ingredients also have different pharmacological activities^4^.

In the *Rosaceae* family, *Chaenomeles* has close phylogenetic relationships with the *Cydonia*, *Malus* and *Pyrus* genera. Lindley established the *Chaenomeles* genus in 1822. Subsequently, some taxonomists began to reclassify some species into this genus. In 1890, Koehne reclassified *Cydonia sinensis (C. sinensis)* as *Chaenomeles sinensis* (Thouin) Koehne (*C. sinensis*)^5^. In 1906, Schneider reclassified *Cydonia cathayensis* (*C. cathayensis*) as *Chaenomeles cathayensis* (Hemsl.) Schneid. (*C. cathayensis*)^6^. In 1929, Nakai also reclassified *Cydonia speciosa* (*C. speciosa*) as *Chaenomeles speciosa* (Sweet) Nakai (*C. speciosa*)^7^. All three of these species had been thought to be members of *Cydonia* before that time. However, Koehne's classification result is still controversial, and not all taxonomists are in agreement^8^. Some taxonomists established independent genera *Pseudochaenomeles* Carr. (1882) and *Pseudocydonia* Schneid. (1906). Phipps^9,10^ considered *Pseudocydonia* an independent genus; in addition, it may be an intermediate type between *Chaenomeles* and *Cydonia*. Due to these controversies, the number of *Chaenomeles* is still ambiguous. According to I. et al^1^, there are only four species in this genus. However, five species have been identified in China, including *Chaenomeles speciosa* (Sweet) Nakai (*C. speciosa*), *Chaenomeles cathayensis* (Hemsl) Schneider (*C. cathayensis*), *Chaenomeles japonica* (Thunb) Lindley (*C. japonica*), *Chaenomeles thibetica* Yu (*C. thibetica*) and *Chaenomeles sinensis* (Thouin) Koehne (*C. sinensis*)^2,11^.

Efficient methods to clarify the taxonomic status of both wild and cultivated germplasms are needed^1^. At present, both morphological traits and various molecular markers are used to solve the taxonomic confusion^12–14^. In the past several years, restriction fragment length polymorphisms (RFLPs), random amplified polymorphic DNAs (RAPDs), amplified fragment length polymorphisms (AFLPs), simple sequence repeats (SSRs), EST-based microsatellites (EST-SSRs), intersimple sequence repeat (ISSRs), sequence characterized amplified regions (SCARs) and single nucleotide polymorphisms (SNPs) have been integrated into the mainstream method to detect evolutionary relationships in related species at the genomic level. Many related studies were reported. In *Chaenomeles,* Barthish et al.^15^ employed RAPDs to analyse offspring families of *C. cathayensis* and *C. speciosa*. Their results showed that the RAPD-based proportion of between-family variability was much higher in the hybrid populations than in pure species. He et al.^16^ evaluated genetic relationships among 52 *C. speciosa* accessions grown in China by combining AFLPs with leaf morphology characteristics; 208 polymorphic markers were identified. Zhang et al. used EST-SSR markers of *Malus* to research the genetic diversity of 33 *Chaenomeles* germplasms and obtained exciting results^17^. With the development of next-generation sequencing (NGS) technologies and algorithms for data processing, RNA-seq has become an efficient and economical approach for genomic and transcriptomic resource mining and has been increasingly considered an efficient tool to identify transcript-wide molecular markers and to solve phylogenetic relationships in non-model species^18–21^. Although few studies have examined transcriptome datasets for individual species, increasing attention has been given to comparative transcriptomes in the genus *Chaenomeles*.

Here, we sequenced the transcriptomes of 10 *Chaenomeles* germplasms belonging to five *Chaenomeles* species and one *D. delavayi* germplasm, which is an outgroup species of *Chaenomeles,* by the Illumina HiSeq 2500 platform. After de novo assembly and functional annotation, common orthologous genes among the 11 germplasms were obtained. We selected single-copy genes from all common orthologous genes to construct a phylogenetic tree to confirm their evolutionary relationship. By pairwise comparison of the orthologous sequences, genes involved in speciation that were under positive selection were obtained to illustrate some key genes during the evolution of this species. Many SSR sites were identified as potential molecular markers for *Chaenomeles*. Common genes containing SSR sites among the 10 *Chaenomeles* germplasms were selected to create a gene expression model in the secondary metabolism pathway. Finally, we developed some SSR markers for these 10 *Chaenomeles* germplasms. This study displays the first comparative exploration of *Chaenomeles* transcriptomes using high-throughput RNA-seq and provides an important methodology and database resources for facilitating further studies on the phylogeny of *Chaenomeles* and its related genus.

## Results

### De novo assembly and unigene qualification results of the 11 germplasms

**Figure 1:**
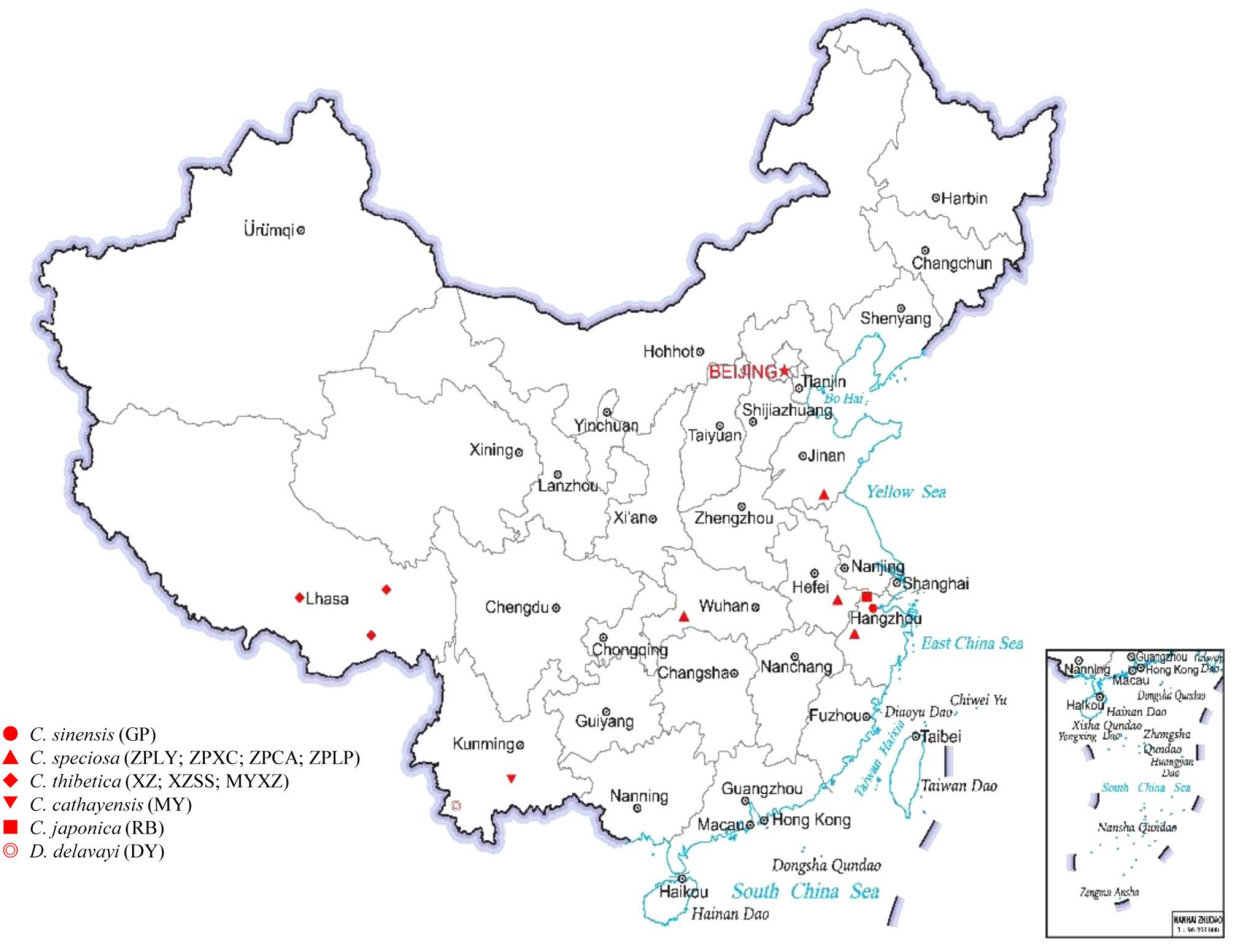
Distribution of the 11 germplasms used for the research in China. Different symbols represent different species. Each germplasm was labelled with specimen IDs as follow: *C. sinensis* (GP) (SWH190512), *C. speciosa* (ZPLY) (SWH190535), *C. speciosa* (ZPXC) (SWH190621), *C. speciosa* (ZPCA) (SWH190657), *C. speciosa* (ZPLP) (SWH190645), *C. thibetica* (XZ) (SWH190805), *C. thibetica* (XZSS) (SWH190823), *C. thibetica* (MYXZ) (SWH190837), *C. cathayensis* (MY) (SWH190711), *C. japonica* (RB) (SWH190527), and *D. delavayi* (DY) (SWH190726). All of the germplasms were identified by Professor J.M.J. The map was downloaded on https://www.tianditu.gov.cn/.

**Figure 2:**
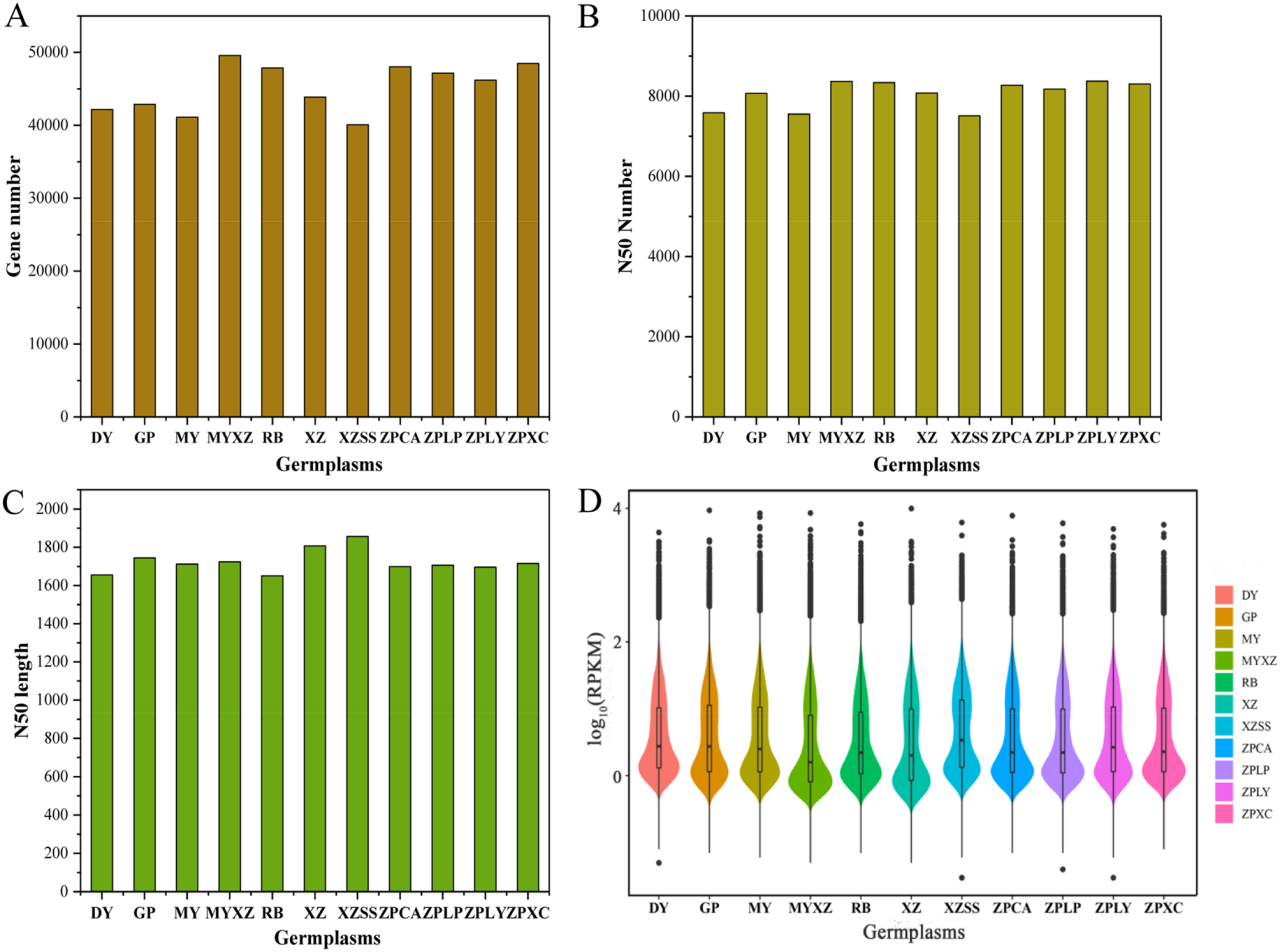
Assembling results and gene expression distribution in 10 germplasms of *Chaenomeles* and *Docynia delavayi* (*D. delavayi*). In this graph, A represents the gene numbers in the 11 materials; B represents the N50 numbers in the 11 materials; C represents the N50 length in the 11 materials; D is a violin graph of the gene expression distribution in the 11 materials. On the X-axis, DY represents *D. delavayi* (DY), GP represents *C.sinensis* (GP), MY represents *C. cathayensis* (MY),XZ represents *C. thibetica* (XZ), MYXZ represents *C. thibetica* (MYXZ), XZSS represents *C. thibetica* (XZSS), RB represents *C. japonica* (RB), ZPCA represents *C. speciosa* (ZPCA), ZPLP represents *C. speciosa* (ZPLP), ZPLY represents *C. speciosa* (ZPLY), and ZPXC represents *C.speciosa* (ZPXC). In violin graph, different colours represent different germplasms. The area and width of the violin graph represent the expression abundance of the expressed genes in the corresponding germplasms. The expression levels in vertical coordinates are standardized by the log_10_ (RPKM) algorithm. The histograms were visulized by Origin pro 2016, violin graph was visulized by R 3.6.1.

11 germplasms including 10 *Chaenomeles* and one related species were collected from 6 provinces of China (Fig. 1). After assembling the unigenes for the 11 germplasms, we obtained 42,169 unigenes in *D. delavayi* (DY), 42,868 unigenes in *C. sinensis* (GP), 48,011 unigenes in *C. speciosa* (ZPCA), 48,487 unigenes in *C. speciosa* (ZPXC), 47,160 unigenes in *C. speciosa* (ZPLP), 46,193 unigenes in *C. speciosa* (ZPLY), 43,875 unigenes in *C. thibetica* (XZ), 40,084 unigenes in *C. thibetica* (XZSS), 49,571 unigenes in *C. thibetica* (MYXZ), 41,091 unigenes in *C. cathayensis* (MY), and 47,856 unigenes in *C. japonica* (RB) (Fig. 2A). The N50 values were 7,584 in *D. delavayi* (DY), 8,069 in *C. sinensis* (GP), 8,273 in *C. speciosa* (ZPCA), 8,304 in *C. speciosa* (ZPXC), 8,174 in *C. speciosa* (ZPLP), 8,374 in *C. speciosa* (ZPLY), 8,077 in *C. thibetica* (XZ), 7,508 in *C. thibetica* (XZSS), 8,369 in *C. thibetica* (MYXZ), 7,552 in *C. cathayensis* (MY), and 8,338 in *C. japonica* (RB) (Fig. 2B).

The N50 lengths were 1,655 bp in *D. delavayi* (DY), 1,744 bp in *C. sinensis* (GP), 1,699 bp in *C. speciosa* (ZPCA), 1,716 bp in *C. speciosa* (ZPXC), 1,706 bp in *C. speciosa* (ZPLP), 1,696 bp in *C. speciosa* (ZPLY), 1,807 bp in *C. thibetica* (XZ), 1,857 bp in *C. thibetica* (XZSS), 1,724 bp in *C. thibetica* (MYXZ), 1,712 bp in *C. cathayensis* (MY), and 1,651 bp in *C. japonica* (RB) (Fig. 2C). These results suggested that our assembly results are good enough for further analyses. In addition, we determined the gene expression levels of the 11 germplasms (Fig. 2D). After standardization with the log_10_ (RPKM) algorithm, the expression levels ranged from − 0.5 to 2. Except for *C. thibetica* (XZ) and *C. thibetica* (MYXZ), the remaining genera had a higher proportion of genes expressed at 0.1–0.2 after standardization. The median gene expression level was greater in *C. thibetica* (XZSS) than in the remaining germplasms (Fig. 2D), indicating different gene expression distributions in *C. thibetica* (XZ), *C. thibetica* (XZSS) and *C. thibetica* (MYXZ).

### Screening of orthologous genes and single-copy genes

**Figure 3:**
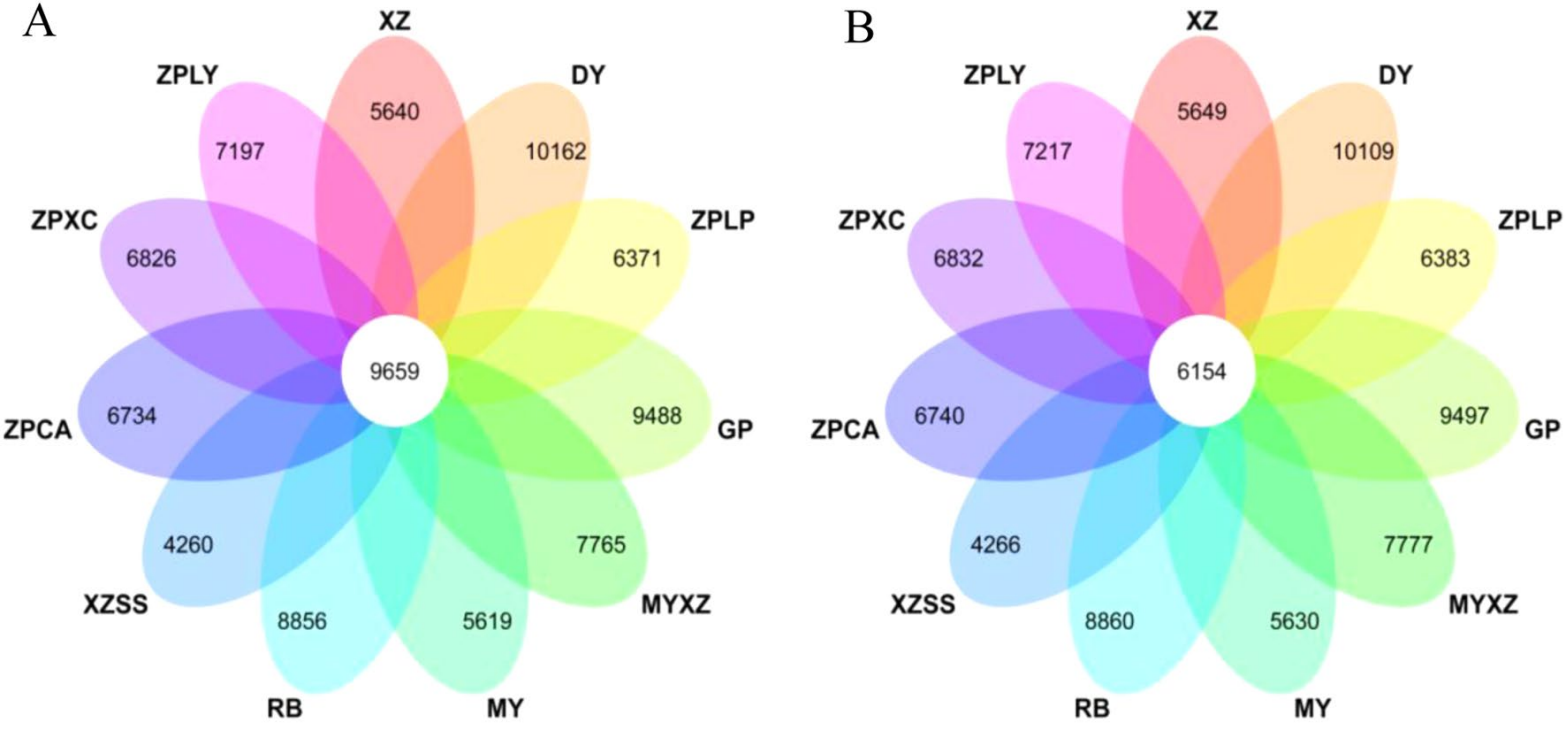
Venn diagrams for the selection of common genes and common single-copy genes among 10 germplasms of *Chaenomeles* and *D. delavay*. A shows the selection of common genes in the 11 materials, B shows the selection of common single-copy genes in the 11 materials. Only common genes among the 11 materials and specific genes in each germplasm are shown in this graph. The diagrams were visualized on https://www.omicshare.com/tools/.

To better understand the similarities and differences among the 11 germplasms, a comparative analysis of the genes among different germplasms is the most efficient method. Genes from different germplasms with the best blast hits were considered orthologues. All these orthologues were commonly used to generate orthologous pairs. We identified orthologous groups for all predicted nucleic acid and protein sequences from the 11 germplasms by using OrthoMCL software. After merging genes from the same family, we finally obtained 28,181 OrthoMCL genes in *C. thibetica* (XZ), 28,908 OrthoMCL genes in *D. delavayi* (DY), 29,882 OrthoMCL genes in *C. speciosa* (ZPLP), 28,725 OrthoMCL genes in *C. sinensis* (GP), 31,682 OrthoMCL genes in *C. thibetica* (MYXZ), 27,989 OrthoMCL genes in *C. cathayensis* (MY), 31,472 OrthoMCL genes in *C. japonica* (RB), 26,214 OrthoMCL genes in *C. thibetica* (XZSS), 30,404 OrthoMCL genes in *C. speciosa* (ZPCA), 30,410 OrthoMCL genes in *C. speciosa* (ZPXC), and 29,960 OrthoMCL genes in *C. speciosa* (ZPLY) (Supplementary Table S1). Then, we identified the common orthologues among the 11 germplasms by a Venn diagram. A total of 9,659 orthologous genes were obtained (Fig. 3A, Supplementary Table S2A). Single-copy genes are valuable resources for phylogenetic analysis and SSR marker selection and only have one copy in the genome of the corresponding species. We also selected single-copy genes out of 9,659 orthologues by using a Venn diagram, and 6,154 orthologous single-copy genes were obtained (Fig. 3B, Supplementary Table S2B).

### Phylogenetic analysis and divergence time estimation based on single-copy orthologous families

**Figure 4:**
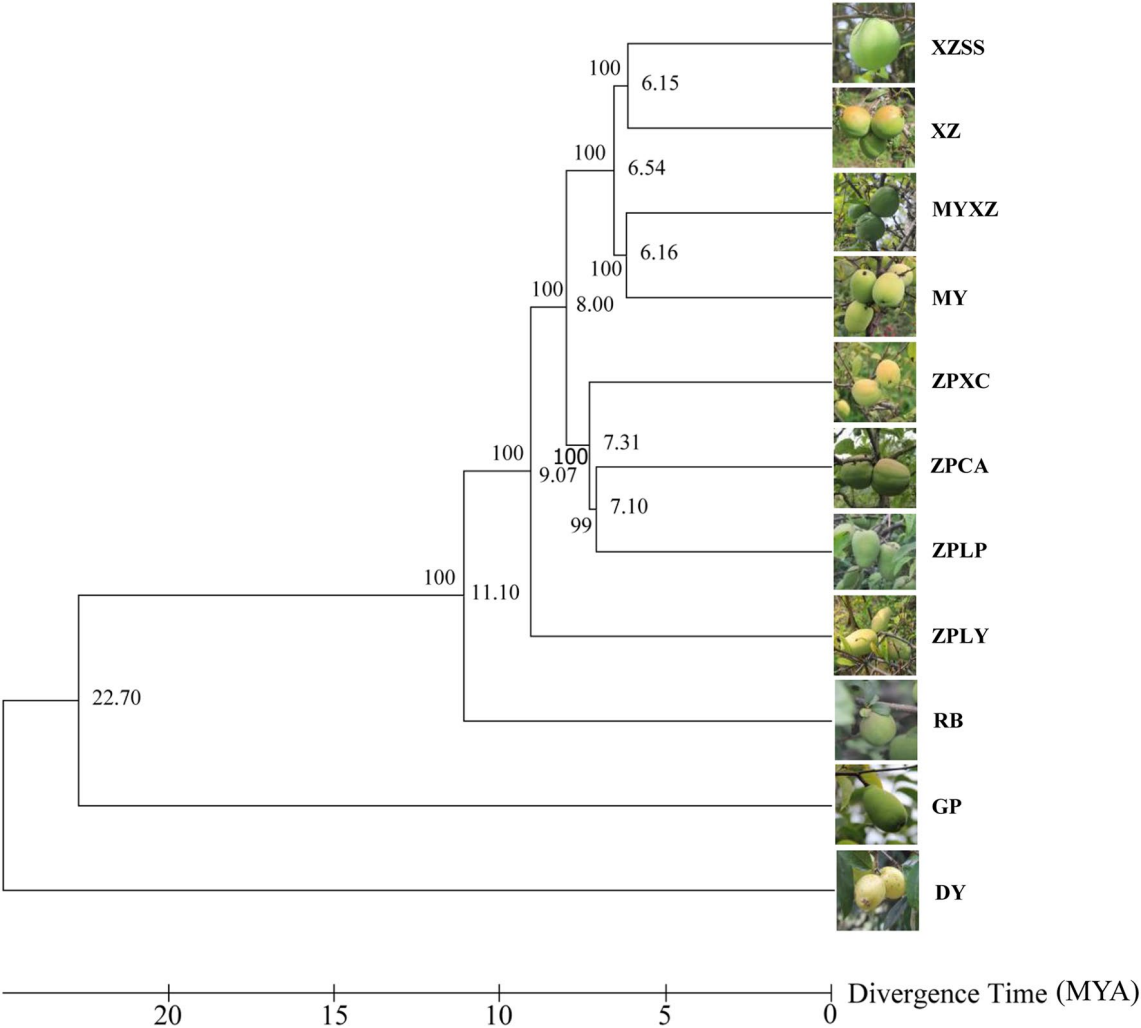
Phylogenetic tree based on common single-copy genes of the 11 germplasms. Numbers to the left of the nodes are bootstrap values; numbers to the right of the nodes are divergence times. MYA is short for million years ago. Evolutionary tree were conducted in MEGA X (V10.02).

In our study, single-copy gene families were used to conduct phylogenetic analysis. According to the evolutionary tree, the 11 germplasms were mainly divided into 5 different clades (Fig. 4). All 11 germplasms shared a common ancestor 22.7 million years ago. After this time point, *D. delavayi* (DY) and *C. sinensis* (GP) began to independently diverge from the remaining nine *Chaenomeles*. Approximately 11.1 million years ago, *C. japonica* (RB) also diverged from the remaining *Chaenomeles*. Herein, *C. japonica* (RB), as a single branch, is also supported by AFLP studies^22^ showing interspecific differentiation. *C. speciosa* (ZPLY), commonly known as ‘Yizhoumugua’ in Linyi City of Shandong Province, is generally considered to originate from interspecific hybridization with other species in the genus^23^. Our study showed that it split from other *Chaenomeles* approximately 9.07 million years ago and had a relatively distant genetic relationship with the other three *Chaenomeles* germplasms, *C. speciosa* (ZPXC), *C. speciosa* (ZPCA) and *C. speciosa* (ZPLP) (Fig. 4). Approximately 8 million years ago, *C. speciosa* separated from *C. thibetica* and *C. cathayensis*. Three germplasms of *C. thibetica*, namely, XZ, XZSS and MYXZ, clustered in the same branch as *C. cathayensis* (MY), showing a very close phylogenetic relationship (Fig. 4). A previous study^23^ suggested that *C. thibetica* might be a hybrid species of *C. cathayensis* and *D. delavayi* (DY). However, more evidence is needed to support this result. *C. speciosa* is the most widely distributed species of the genus in China. Due to its outstanding pharmacological effects, it has been domesticated and cultivated for a long time and has formed three genuine producing areas, corresponding to the distribution of three local varieties, namely, ‘Cunmugua’ (ZPCA), ‘Xuanmugua’ (ZPXC) and ‘Ziqiumugua’ (ZPLP). Our phylogenetic analysis indicated their close relationship.

### GO and KEGG analysis results for single-copy genes in 11 germplasms

**Figure 5:**
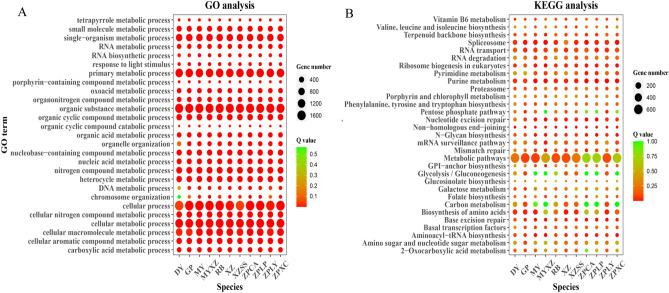
GO and KEGG analyses for common single-copy genes among 10 germplasms of *Chaenomeles* and *D. delavayi*. In this graph, the pot size indicates the number of enriched gene; the degree of significance decreases from red to green. In both the GO and KEGG analysis results, only the top15 GO terms and pathways are shown. The graphs were visualized by R 3.6.1.

To understand the function of 6,154 common single-copy genes in 11 germplasms, we conducted GO and KEGG analyses (Fig. 5). According to the GO and KEGG results, these 6,154 genes shared similar metabolic processes. After merging the top 15 GO terms in each germplasm, we obtained 27 common GO terms (Fig. 5A). Common single-copy genes were significantly enriched in these 27 GO metabolic processes in all 10 *Chaenomeles* germplasms except *D. delavayi* (DY). In *D. delavayi* (DY), common single-copy genes were insignificantly enriched in DNA metabolic process (GO: 0006259) with a *p*. adjust of 0.294 and chromosome organization (GO: 0051276) with a *p*. adjust of 0.564, indicating its different metabolic process from *Chaenomeles* (Fig. 5A Supplementary Table S3). All 11 germplasms were especially enriched in nitrogen compound metabolic process (GO: 0006807), organic cyclic compound metabolic process (GO: 1901360), heterocycle metabolic process (GO: 0046483), cellular nitrogen and compound metabolic process (GO: 0,034,641), and nucleic acid metabolic process (GO: 0090304) (Fig. 5A, Supplementary Table S3).

In the KEGG analysis, after merging the top 15 pathways in each germplasm, we finally obtained 31 common pathways (Fig. 5B). The common single-copy genes were significantly enriched in nonhomologous end-joining (ko03450), ribosome biogenesis in eukaryotes (ko03008) and purine metabolism (ko00230) in all 11 germplasms (Fig. 5B Supplementary Table S4). In addition to these two pathways, *D. delavayi* (DY) was also enriched in biosynthesis of amino acids (ko01230) with a gene number of 97 out of 297 (*Q* value 0.032). *C. sinensis* (GP) was also enriched in spliceosome (ko03040) and biosynthesis of amino acids (ko01230) with gene numbers of 89 out of 263 (*Q* value 0.008) and 96 out of 296 (*Q* value 0.0158), respectively. *C. cathayensis* (MY) was enriched in aminoacyl-tRNA biosynthesis (ko00970) with a gene number of 31 out of 70 (0.006). *C. thibetica* (MYXZ) was enriched in base excision repair (ko03410) with a gene number of 24 out of 52 (*Q* value 0.0006). *C. japonica* (RB) was also enriched in N-glycan biosynthesis (ko00510) with a gene number of 24 out of 57(*Q* value 0.0257). *C. thibetica* (XZ) was also significantly enriched in biosynthesis of amino acids (ko01230) and nucleotide excision repair (ko03420) with gene numbers of 97 out of 290 (*Q* value 0.0097) and 36 out of 87 (*Q* value 0.0097), respectively. *C. thibetica* (XZSS) was enriched in base excision repair (ko03410) and spliceosome (ko03040) with gene numbers of 24 out of 48(*Q* value 0.0204) and 88 out of 247(*Q* value 0.0204), respectively. *C. speciosa* (ZPCA) was significantly enriched in nucleotide excision repair (ko03420) with a gene number of 36 out of 89 (*Q* value 0.0014). Both *C. speciosa* (ZPLP) and *C. speciosa* (ZPLY) were enriched in base excision repair (ko03410) with gene numbers of 25 out of 54 (*Q* value 0.0016) and 25 out of 46 (*Q* value 0.00096), respectively. *C. speciosa* (ZPXC) was enriched in aminoacyl-tRNA biosynthesis (ko00970) and spliceosome (ko03040) with gene numbers of 32 out of 70 (*Q* value 0.0004) and 88 out of 290 (*Q* value 0.0034), respectively (Fig. 5B, Supplementary Table S4).

### Detecting genes under selective pressure

**Figure 6:**
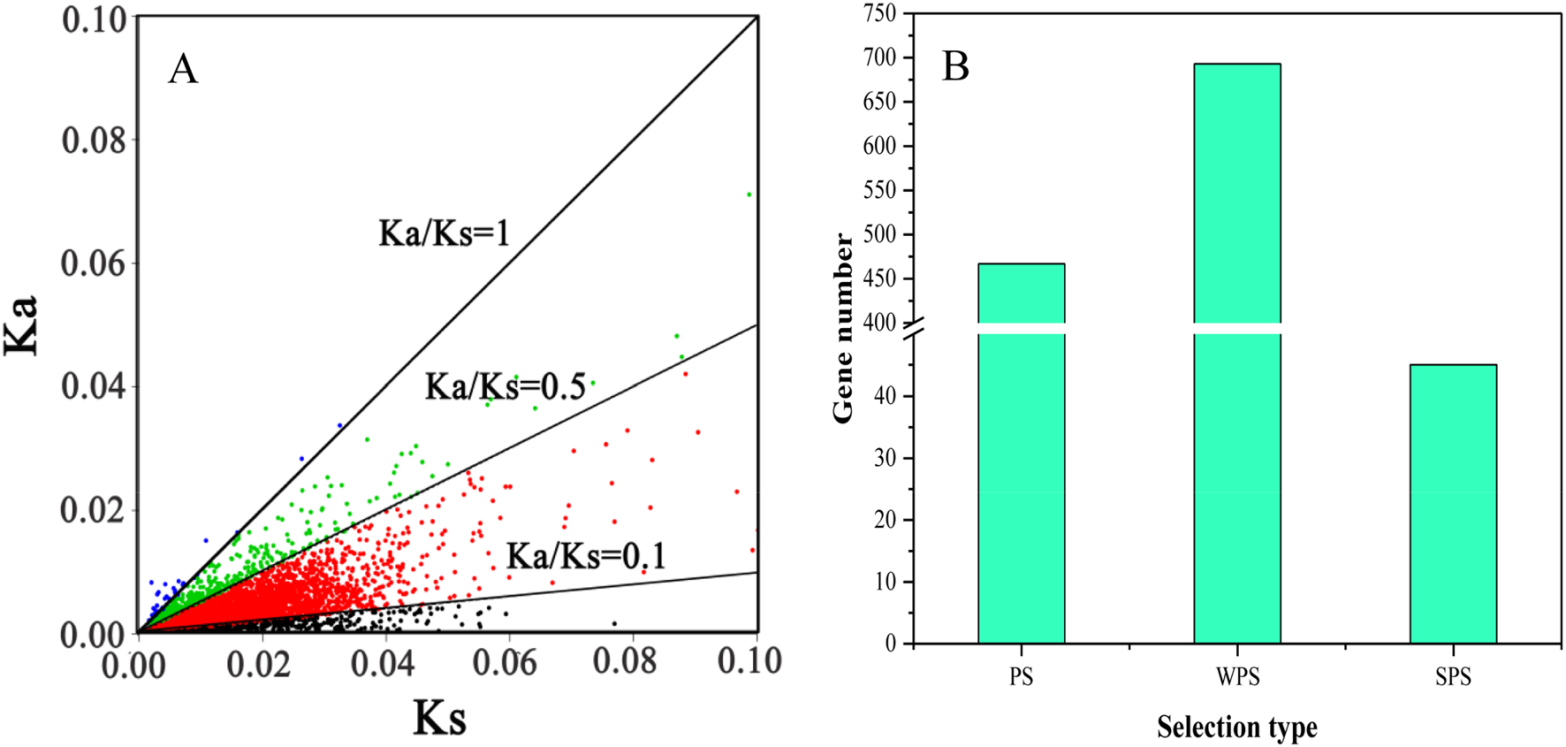
Selection pressure analyses for single-copy genes in common genes of 10 germplasms of *Chaenomeles* and *D. delavayi*. In graph A, each coloured dot represents one single-copy gene family. The top diagonal line represents Ka/Ks = 1; the first dotted line represents Ka/Ks = 0.5; and the second dotted line represents Ka/Ks = 0.1. In graph B, PS is short for purification selection; WPS is short for weak positive selection; and SPS is short for strong positive selection.Scatter diagram was visualized by R 3.6.1. Histogram was visulized by Origin pro 2016.

The nonsynonymous substitution rate (Ka) and synonymous substitution rate (Ks) were used to estimate the change in coding protein sequences. The magnitude of the Ka/Ks ratio provides evidence of genes under strong functional constraints (Ka/Ks < 1) or undergoing adaptive evolution (Ka/Ks > 1)^24–26^. The ratio of the nonsynonymous substitution rate (Ka) and synonymous substitution rate (Ks) were used to assess whether genes were under selection. After filtering, a total of 6057 genes remained for further analysis. According to the BLASTn results, a total of 6057 single-copy genes underwent selective processing (Fig. 6A, Supplementary Table S5A). Among them, 467 out of 6057 genes underwent purification selection, indicating that these genes are disadvantageous during the evolutionary process. A total of 693 out of 6,057 genes experienced weak positive selection, and 45 out of 6,057 genes experienced strong positive selection, indicating that those genes are domain genes that determine the direction of evolution (Fig. 6B, Supplementary Table S5B-D). Furthermore, we blasted these 45 positively selected genes to *Arabidopsis* (Supplementary Table S5D). Nineteen out of 45 genes had homologous genes in *Arabidopsis*. ORTHOMCL10413 (*RGA2*) mainly acts as a repressor of the gibberellin (GA) signalling pathway through a transcription coactivator of the zinc finger transcription factors GAF1/IDD2 and ENY/IDD1 to regulate gibberellin homeostasis and signalling^27^. ORTHOMCL9348 (*RPL5*) participates in cell proliferation and plays a role in translation in leaf dorsoventral patterning to specify leaf adaxial identity^28^. ORTHOMCL8960 (*HEL*) has a modular structure consisting of an N-terminal hevein-like domain (CB-HEL) and a C-terminal domain (CD-HEL) that is post-translationally processed. Both domains show strong antifungal activity, indicating that this gene is responsible for defending plants against pathogen attack^29^. ORTHOMCL9718 (*FIP2*) can delay flowering by repressing the expression of FLOWERING LOCUS C^30^. ORTHOMCL4564 (*CSA1*) also plays an important role in disease defence^31^. ORTHOMCL9773 (*MYB27*) is a member of the MYB family, and many studies have indicated that MYB family members can regulate secondary metabolism, control cell shape, defend against disease and increase hormone responses^32^. ORTHOMCL8338 (*BZIP60*) is involved in controlling endoplasmic reticulum pressure to enhance the immune response in both animals and plants^33^. In addition, although there are still no reports on the function of ORTHOMCL5656 (*CYS6*), ORTHOMCL6947 (*HPR3*) and ORTHOMCL6257 (*F11P17.9*) in plants, according to the GO analysis results, these three genes mainly participated in the disease defence process (Supplementary Table S3). All of these results indicate that most of the positively selected genes are involved in building the plant disease defence system. Thus, these plants can survive long-term evolution and selection processes.

### Model of gene expression for genes containing simple sequence repeats (SSRs) in the biosynthesis of secondary metabolic pathways of 10 *Chaenomeles* germplasms

**Figure 7:**
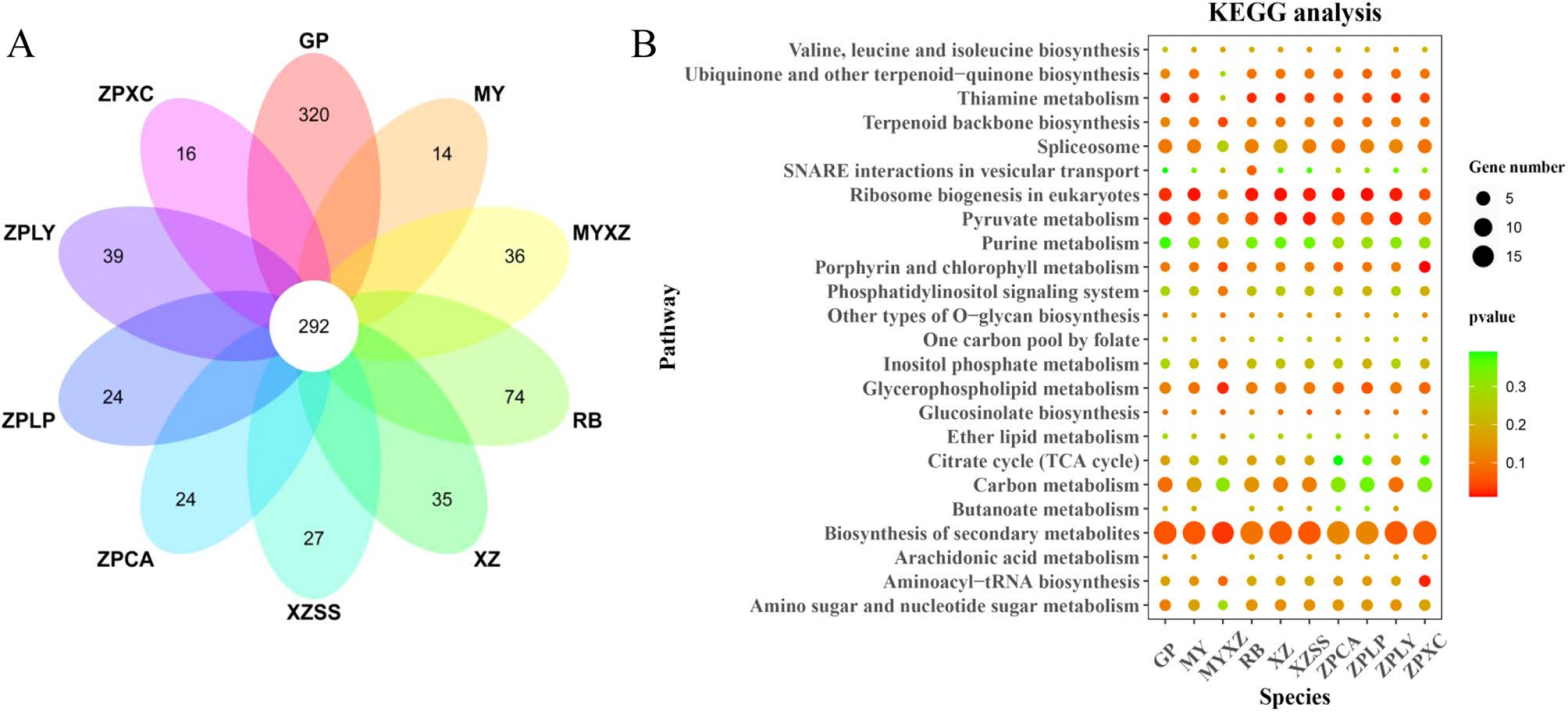
Venn diagram and KEGG analysis for common single copy genes in 10 germplasms of *Chaenomeles* containing SSR sites. In graph B, the dot size indicates the number of enriched gene; the degree of significance decreases from red to green.Venn diagram was visualized on https://www.omicshare.com/tools/. KEGG result was visulized by R3.6.1.

**Figure 8:**
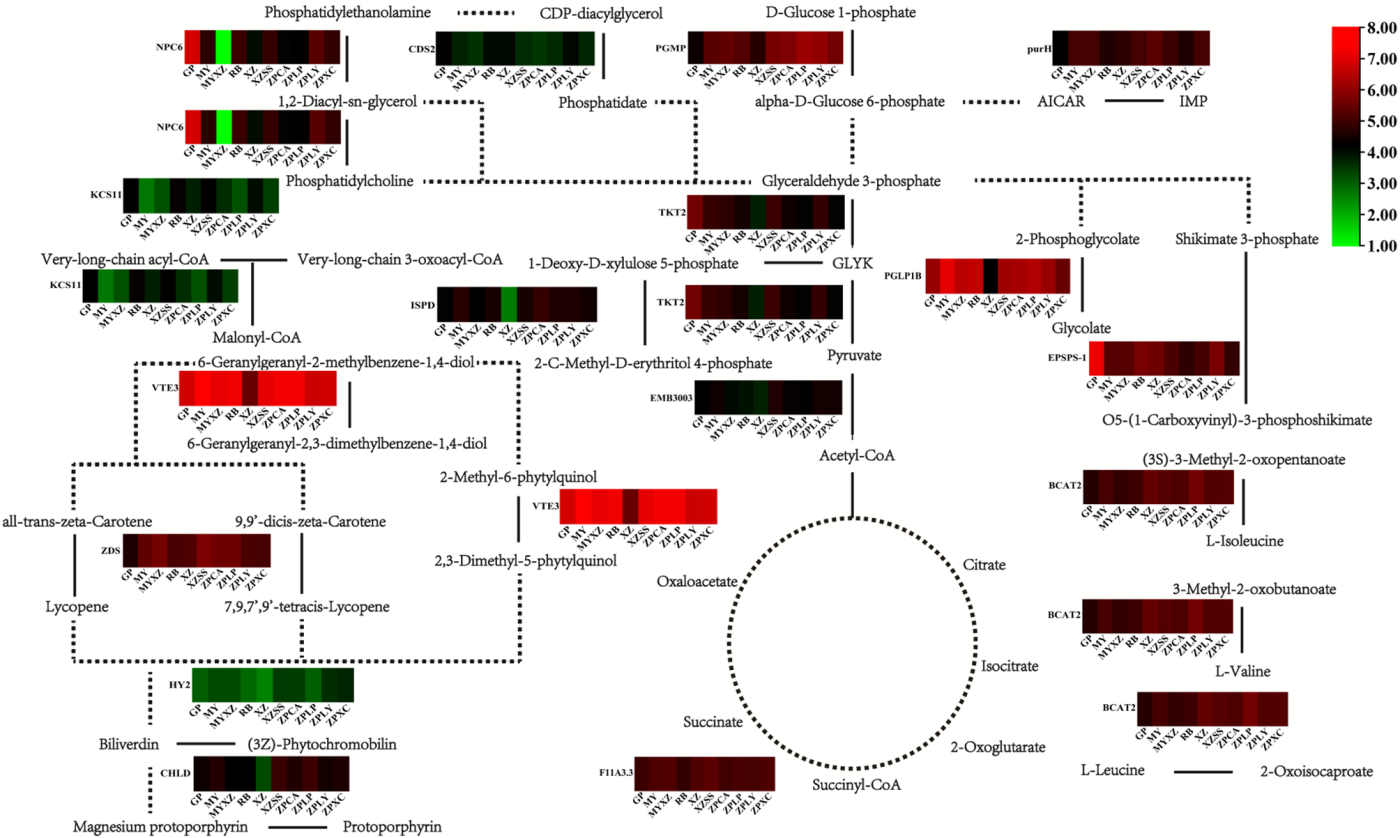
Gene expression models in biosynthesis of secondary metabolites pathway. This graph displays part of the biosynthesis of secondary metabolites. Dotted lines describe indirect metabolism flow; solid lines describe direct metabolism flow. The coloured bar describes the gene expression level in the corresponding position. RPKMs in heatmaps are standardized by the log_2_ (RPKM) algorithm; green indicates a low expression level, and red indicates a high expression level. Metabolites pathway was drawn by Adobe Illustrator CS5 software, heatmaps were visualized by TBtools 0.6669.

We predicted the SSR sites in all 6,154 common single-copy genes by using MISA software. A total of 1,210, 1,151, 1,128, 1,253, 1,186, 1,225, 1,178, 1,181, 1,170, and 1,165 genes contained SSR sites in *C. sinensis* (GP), *C. japonica* (RB), *C. cathayensis* (MY), *C. thibetica* (XZ), *C. thibetica* (XZSS), *C. thibetica* (MYXZ), *C. speciosa* (ZPCA), *C. speciosa* (ZPLP), *C. speciosa* (ZPXC), and *C. speciosa* (ZPLY), respectively (Supplementary Table S6). Five SSR types, di-, tri-, tetra-, penta- and hexanucleotides, were counted. Among them, di- and trinucleotides were the main repeat units, ranging from 60.43% to 63.69% and 28.72% to 30.89%, respectively (Supplementary Table S7A-J). Then, we selected the common genes that contained SSR markers for 10 germplasms. In total, 292 common single-copy genes were selected (Fig. 7, Supplementary Table S8). Furthermore, we conducted KEGG analyses for these 292 common single-copy genes. All 10 germplasms were significantly enriched in the biosynthesis of secondary metabolites (Supplementary Table S9). Therefore, we examined this pathway to determine the gene expression model for each germplasm. Although all 10 germplasms had genes participating in the same process in the pathway, there still existed differences in the gene expression level. For examples, from phosphatidylethanolamine to 1,2-diacyl-sn-glycerol and 1,2-diacyl-sn-glycerol to phosphatidylcholine, *NPC6* expression was lower in *C. thibetica* (MYXZ) than in the rest of the germplasms. From magnesium protoporphyrin to protoporphyrin, *CHLD* expression was lower in *C. thibetica* (XZ) than in the remaining germplasms. During transformation from 3-oxoacyl-CoA to acyl-CoA and malonyl-CoA, *KCSLL* expression was higher in *C. sinensis* (GP), *C. japonica* (RB), *C. thibetica* (XZSS) and *C. speciosa* (ZPXC) than in the other germplasms (Fig. 8, Supplementary Table S10). However, there were similarities in the gene expression levels in the 10 germplasms. For instances, during the transformation of 6-geranylgerany-2-methylbenzene-1,4-diol to 6-geranylgeranyl-2,3-dimetheyl benzene-1,4-diol, shikimate 3-phosphate to O5-(1-Carboxyvinyl) -3- phosphoshikimate, (3S)-3-methyl-2-oxopentanoate to L-isoleucine, 3-methyl-2-oxobutanoate to L-valine and L-leucine to 2-oxoisocaproate, we detected that *VTE3*, *EPSPS-1* and *BACT2* had a high expression level in all of 10 *Chaenomeles* germplasms (Fig. 8, Supplementary Table S10).

### Primer screening, polymorphism study and reconstruction of an evolutionary tree based on 10 selected SSRs

Then, we designed primers for genes containing SSR sites with Primer 3 following the rules described in the methods. In total, we obtained 373, 357, 362, 366, 355, 354, 352, 365, 370 and 372 primers for *C. sinensis* (GP), *C. cathayensis* (MY), *C. thibetica* (MYXZ), *C. japonica* (RB), *C. thibetica* (XZ), *C. thibetica* (XZSS), *C. speciosa* (ZPCA), *C. speciosa* (ZPLP), *C. speciosa* (ZPLY) and *C. speciosa* (ZPXC), respectively (Supplementary Table S11A). The number of common genes used for primer design in 10 *Chaenomeles* germplasms was 110 (Supplementary Table S11B).

A total of 110 pairs of SSR primers were synthesized, and the 10 *Chaenomeles* germplasms were used to verify the PCR amplification ability. Based on this result, a total of 45 pairs of primers were screened out (Supplementary Table S12). Then, capillary electrophoresis was used to detect the levels of polymorphism and specificity of the 45 primers. The results showed that only 10 pairs of primers had good polymorphism levels. Primers from ORTHOMCL6247, ORTHOMCL8473, ORTHOMCL7263 ORTHOMCL4541, ORTHOMCL9476, ORTHOMCL9834 and ORTHOMCL8700 had the best polymorphism levels (Table 1, Supplementary Fig. S1). The primers from ORTHOMCL4735, ORTHOMCL8947 and ORTHOMCL6535 also had relatively good polymorphism levels but had poor amplification results for individual germplasms (Table 1, Supplementary Fig. S1). Table 1 displayed all 10 selected primers, including tandem repeat units, annealing temperature and detailed forward and reverse primers (Table 1).

**Table 1:** Primer and fragment length for selected genes.

Primer code	Othology ID	Gene symbol	Tandem repeats	Expected length (bp)	Annealing temperature (°C)	Forward primer (5′–> 3′)	Reverse primer (5′–> 3′)
CHSWH1	ORTHOMCL4541	*ORP3C*	(AG)_11_	104	60	TCTTCCCTTTCATTTTCCGA	TCTGACCCTTCTCTGGGCTA
CHSWH2	ORTHOMCL4735	*CRK25*	(TCC)_5_	272	60	AAGGGGGACGAGTTCTGTTT	GTCTCACCACTCGGTTCGTT
CHSWH3	ORTHOMCL9476	*SDAD1*	(TGA)_5_	162	60	AAGATGACGGCAATGAGGAC	CATCTTCGCTTCCACTGTCA
CHSWH4	ORTHOMCL6247	*T19L5.4*	(ACC)_8_	272	60	CTGAACAAACTCACCCCCAT	ATTGAACGCTTGGATAACCG
CHSWH5	ORTHOMCL9834	*KRI1*	(GGAGAG)_4_	256	60	ATTTTGAATCGGACGACGAC	CCATCCTCCATCAAATGCTT
CHSWH6	ORTHOMCL8700	*BBR*	(AGC)_5_	184	60	GAGGACGAAGAAATTGGCAG	ATTTGCAGGTGTAGGGATGC
CHSWH7	ORTHOMCL8947	*trmB*	(CAGCAA)_4_	211	60	GCTGCATTACCCAGAAGAGC	GGTTGAGATTAAGGTCGGCA
CHSWH8	ORTHOMCL6535	*–*	(CT)_9_cactctctcc(CT)_6_	112	60	TCACTTTGGTCCATGTCTGC	CGATATGTGTGTGCTCGGAC
CHSWH9	ORTHOMCL7263	*–*	(GGC)_5_	171	60	CCGTACACAAAACAAGCCCT	ATATTCCCGGAAACTGACCC
CHSWH10	ORTHOMCL8473	*DOF5.3*	(CAC)_8_	123	60	GGACCATGGGCAATAACAAC	GAGAGCCATATGATCCGGG

Polymorphism analysis of these SSR loci showed that the average allele number was 6.6, ranging from 5 to 9. The average observed heterozygosity was 0.243, ranging from 0 to 0.455. The average expected heterozygosity was 0.823, ranging from 0.723 to 0.935 (Table 2). Lower expected heterozygosity than observed heterozygosity suggests a departure from Hardy–Weinberg equilibrium and a high probability of inbreeding^34^. All 10 selected SSRs had lower expected heterozygosity than observed heterozygosity, indicating that inbreeding processes occurred among *Chaenomeles*. Traditional studies have divided the polymorphism information content into three classes: high polymorphism (PIC > 0.5), moderate polymorphism (0.25 < PIC < 0.5) and low polymorphism (PIC < 0.25)^35^. In our research, the PIC values of the 10 selected SSRs were more than 0.5, suggesting high polymorphism and many effective genetic markers from our screened primers.

**Table 2:** Polymorphic information of the 10 SSR loci in the 10 germplasms.

Locus	Na	Ho	He	PIC
CHSWH1	5	0.182	0.801	0.726
CHSWH2	6	0.000	0.837	0.761
CHSWH3	5	0.000	0.833	0.746
CHSWH4	8	0.273	0.810	0.750
CHSWH5	7	0.455	0.831	0.765
CHSWH6	8	0.364	0.866	0.804
CHSWH7	9	0.333	0.935	0.871
CHSWH8	6	0.455	0.810	0.738
CHSWH9	6	0.273	0.723	0.658
CHSWH10	6	0.091	0.784	0.709
Mean	6.6	0.243	0.823	0.753

Na: number of allele; Ho: observed heterozygosity; He: expected heterozygosity; PIC: polymorphism information content.

Then, we used the genes amplified by these 10 primers to build an evolutionary tree. The results showed that the 11 germplasms were mainly divided into two clades (Supplementary Fig. S2). *D. delavayi* (DY), *C. sinensis* (GP), *C. japonica* (RB), *C. speciosa* (ZPLY), *C. speciosa* (ZPLP) and *C. cathayensis* (MY) were clustered in the same clade and diverged sharply from the remaining germplasms. Among them, *D. delavayi* (DY), *C. sinensis* (GP), *C. japonica* (RB) and *C. speciosa* (ZPLY) had a similar evolutionary relationship based on all 6,154 single-copy genes (Fig. 4, Supplementary Fig. S2). Three germplasms from Tibet, *C. thibetica* (MYXZ), *C. thibetica* (XZ) and *C. thibetica* (XZSS), diverged from the above six germplasms, suggesting genetic differentiation between them. The four *C. speciosa* germplasms distributed in different clades may be caused by the influence of different geographical distributions and artificial domestication on these ten screened genes. In total, the evolutionary relationship based on 10 screened genes did not completely correspond with that based on 6,154 single-copy genes*.*

## Discussion

Several approaches, including morphological markers, cytological markers, and biochemical markers, have been explored to classify plant resources^22^. However, each technique is imperfect. For example, great phenotypic differences between individuals from geographically isolated populations create obstacles for taxonomists. Subjective factors can lead to misclassification. The limited number of isoenzymes and low resolution of cytology prevent taxonomists from recognizing many plants. Due to these flaws, molecular markers have become a prevalent tool in evolutionary studies in *Chaenomeles* and related species^36–39^. Various molecular markers have provided reliable and convincing evidence for related studies, promoting phytotaxonomic research, whereas some technologies are still unaffordable because of their high cost. Currently, RNA-seq has become an economical and efficient tool for accessing molecular information.

The phylogeny and classification of *Chaenomeles* have been controversial for several centuries, especially whether *C. sinensis* is a member of *Chaenomeles*. Robertson et al.^40^ examined the phylogenetic relationships among 88 genera of Rosaceae, and the results show that it is far from the *Chaenomeles* group, indicating that *C. sinensis* is not a member of *Chaenomeles*. In our research, we found that *C. sinensis* had a closer evolutionary relationship with *D. delavayi,* confirming Robertson’s result. There was obvious differentiation between *C. sinensis* (GP) and all other species of *Chaenomeles*. In terms of phenotype, *C. sinensis* (GP), which has the characteristics of branches unarmed, flowers solitary, coetaneous, sepals reflexed, stipules ovate-lanceolate and margin glandular serrate, is also significant different from the other *Chaenomeles* species, which have the characteristics of branches armed, flowers fascicled, precocious or coetaneous, sepals erect, rarely reflexed, stipules herbaceous, reinform or auriculate and margin serrate^23^. Pollen morphology also shows that *C. sinensis* (GP) is distant from other species^41^. Therefore, based on the phylogenetic results and phenotypic characteristics, we support that *C. sinensis* should not belong to *Chaenomeles* but should be an independent genus *Pseudocydonia*. This view was also supported by our other study on the phylogeny of *Chaenomeles* based on chloroplast genome sequencing^42^.

The genetic characteristics of populations should be dictated by the interplay of genetic drift, gene flow and natural selection. These processes may be strongly influenced by the demography and spatial distribution of populations^43^. In our study, the same species from different regions exhibited not only differences in appearance but also differences in their main chemical components; for this reason, the term genuine regional drug appeared in traditional Chinese medicine. From a molecular marker perspective, although these germplasms are the same *Chaenomeles*, they have formed genetically diverse offspring leading to different types (Fig. 8), as has been reported in Bartish’s research, which discriminated 42 *Chaenomeles* by the RAPD method^44^.

For closely related taxa, the advantages of homologous single-copy genes for phylogenetic and phylogeographic analysis are clear because of their rapid evolutionary rates and clear avoidance of paralogy^45–48^. According to past studies, many single-copy genes have been identified in many plants, including *Euasterids*, *Rosaceae*, *Poaceae*, *Cycades* and 29 other angiosperms^49–53^. For example, Duarte et al. identified a series of 959 single-copy genes in *A. thaliana*, *P. trichocarpa*, *V. vinifera* and *O. sativa* and used 18 single-copy genes to improve the resolution of *Brassicaceae* phylogeny^45^. In this study, we identified 6,154 single-copy genes according to the strict filtering criterion. All these detected single-copy genes proved highly effective in the phylogenetic reconstruction of *Chaenomeles* species, which provided a rough phylogenetic framework for the whole genus (Fig. 4). Therefore, this result indicated that all these candidate single-copy genes from the transcriptomes of *Chaenomeles* species have good application in molecular phylogeny studies of the *Chaenomeles* genus.

In our results, 10 out of 45 SSR markers were finally confirmed to distinguish the 10 *Chaenomeles* germplasms. They have good polymorphism and amplification levels. The efficiency of the primer development rate was 22.2%, which was slightly lower than that reported by Wang et al. (28.6%), who developed SSR markers in pineapple. For the development rate, many factors can influence the results, such as the selected length of the primers, the genetic diversity of the tested species, and development methods. For instance, Wang^54^ compared four methods, including ISSR, SSR, SCoT and RAPD, by using 47 germplasms of *Ananas comosus* (L.) Merr. Among them, the development rate of RAPD reached 47.06%, which was significantly higher than that of the other methods. However, the SCoT and SSR methods had good comprehensive performance compared to the two other methods. Taken together, our results have proven that SSR marker development based on the transcriptome is promising.

Although classification studies in *Chaenomeles* have made great progress, nomination and classification systems still have some problems. The results of this study support the idea of *C. sinensis* as an independent genus, *Pseudochaenomeles*. With increasing exploration of its edible and medicinal value, it is important to distinguish the various *Chaenomeles* to avoid misuse. Thus, the determination of authoritative criteria is urgent. Due to the delay in the sequencing of the *Chaenomeles* genome, it is still difficult to explore SSR markers efficiently. Because of the low cost of transcriptome sequencing, this method will be the best choice; thus, our research provides a new methodology and reference for future related research in *Chaenomeles*. The high genetic diversity in the genus *Chaenomeles,* as inferred from molecular markers, is also useful for improvement, breeding and selection projects in related species.

## Methods

### Plant material, total RNA extraction and Illumina sequencing

All five species of *Chaenomeles* and one related species (*D*. *delavayi*) were collected (Fig. 1). *C*. *sinensis* (GP) was collected in Anji County of Zhejiang Province. According to the local common name, *C*. *speciosa* was divided into four types, including *C*. *speciosa* ‘Chunmugua’ (ZPCA) from Chunan County of Zhejiang Province, *C*. *speciosa* ‘Ziqiumugua’ (ZPLP) from Changyang County of Hubei Province, *C*. *speciosa* ‘Xuanmugua’ (ZPXC) from Xuancheng City of Anhui Province and *C*. *speciosa* ‘Yizhoumugua’ (ZPLY) from Linyi City of Shandong Province. Three local types of *C*. *thibetica* were also collected and distributed in Lhasa (XZ), Bomi (XZSS) and Motuo (MYXZ) of Tibet. *C*. *cathayensis* (MY) was collected from Shiping County, Yunnan Province. *C*. *japonica* (RB) was introduced from Japan and collected from the Germplasm Resource Bank of *Chaenomeles* in Anji County of Zhejiang Province. *D*. *delavayi* (DY) was collected from Lancang County of Yunnan Province. The voucher specimens were deposited in the Herbarium of Research of Institute of Subtropical Forestry, Chinese Academy of Forestry with the following specimen IDs: *C. sinensis* (GP) (SWH190512), *C. speciosa* (ZPLY) (SWH190535), *C. speciosa* (ZPXC) (SWH190621), *C. speciosa* (ZPCA) (SWH190657), *C. speciosa* (ZPLP) (SWH190645), *C. thibetica* (XZ) (SWH190805), *C. thibetica* (XZSS) (SWH190823), *C. thibetica* (MYXZ) (SWH190837), *C. cathayensis* (MY) (SWH190711), *C. japonica* (RB) (SWH190527), and *D. delavayi* (DY) (SWH190726). All of the germplasms were identified by Professor J.M.J.

A total of 15 duplicate leaves were collected from each germplasm resource. According to the manufacturer’s instructions, total RNA was isolated from mature leaf samples as described in Owczarek et al.^55^. After testing the qualification of extracted RNA, including concentration, RIN value, ratio of 28S to 18S, and OD_260_ to OD_280_ by using an Agilent 2100 Bioanalyser and a NanoDrop, the RNAs from the same germplasm were pooled to prepare a cDNA library by using the cDNA Synthesis Kit (Illumina Inc., San Diego, CA) following the manufacturer’s recommendations^56^. Finally, the constructed cDNA libraries were sequenced using an Illumina HiSeq 2500 by a paired-end 150 bp strategy.

### Filtering and assembly of the reads, quantitative calculation and functional annotation for unigenes

First, raw reads were filtered with fastp software (**V0.19.4**) by removing adaptors and low-quality reads^57^. Then, the cleaned reads were de novo assembled using Trinity with the default parameters for each individual sample^58^. The expression level of unigenes was calculated by RSEM^59^ software (**V1.3.0**) and standardized by RPKM. Unigenes were queried against the nonredundant protein (Nr) database, the Swiss-Prot protein database, the Kyoto Encyclopedia of Genes and Genomes (KEGG) pathway database and the Cluster of Orthologous Groups (COG) database using BLASTx with an *E*-value cut-off of 1e−5^60^. According to the BLAST results, the coding region sequences (CDSs) of unigenes were extracted and translated into peptides. For the unigenes without matches in the above four databases, we predicted the coding region sequences by using TransDecoder. Gene Ontology (GO) annotation of the unigenes was obtained using Blast2GO^61^. Enrichment analyses of GO terms and KEGG pathways were conducted as described in Li et al.^62^.

### Identification of orthologues, single-copy genes and multiple-copy genes

Diamond (V3.1)^63^ and OrthoMCL (V2.0.9)^64^ software were used to identify the orthologues among the 11 germplasms. In the first step, multiple sequence comparisons among different germplasms were conducted with cut-offs of *E*-value ≤ 1e−5 and query cover ≥ 30% by using Diamond. In the second step, the orthologues were merged into different families by using OrthoMCL. These obtained orthologous genes can be divided into single-copy genes and multiple-copy genes according to their copy numbers.

### Phylogenetic analysis and selective pressure analysis

Based on the above analyses, we first constructed sequence alignment by using MUSCLE (multiple sequence alignment with high accuracy and high throughput, V3.8.31) for single-copy genes. Then, a timetree was estimated by the RelTime method^65,66^ with the maximum likelihood method^67^. Evolutionary analyses were conducted in MEGA X^68^. To detect whether genes were affected by natural conditions, Paml-codeml was used to compute the nonsynonymous substitution rate (Ka), synonymous substitution rate (Ks) and ratio of Ka to Ks. To avoid influences from nonsignificant or excessive variation, these single-copy genes were discarded as described by Goodman et al.^69^. We aligned orthologous pairs^70^. Orthologous genes with a Ka/Ks > 1 were considered genes under strong positive selection; orthologous genes with 0.5 < Ka/Ks < 0.1 were considered affected by weak positive selection; and orthologous genes with a Ka/Ks < 0.1 were considered affected by negative selection. The significance tests were measured with *p* values, which were corrected via the false discovery rate (FDR).

### Expression model of single-copy genes in the secondary metabolite pathway

After performing KEGG analysis, a significantly enriched pathway, the secondary metabolite pathway, was selected for detecting a gene expression model among the 10 *Chaenomeles* germplasms. Genes in key steps of the secondary metabolite pathway were obtained according to web-version map01110. The pathway was depicted by Adobe Illustrator CS5 software^71^, and heatmaps were visualized by TBtools 0.6669^72^.

### Identification, SSR screening, polymorphism study of SSR loci and reconstruction of phylogenetic tree among 10 *Chaenomeles* germplasms

We identified SSR motifs in all unigenes using MISA software^73^. Based on tandem repeats, including AAC, ACA, CAA, GTT, TGT and TTG, all these SSR motifs were divided into di-, tri-, tetra-, penta- and hexanucleotide types. PCR primers were designed for the SSRs using the program Primer3; they were designed to have more than 150 bp flanking sequences^74^. All of the primers used for SSR development are shown in Supplementary Table S12.

DNA was extracted from the leaves of the 11 germplasms as described in Bartish et al.^75^. Fluorescently labelled primers were synthesized for amplifying gene fragments based on the following rules: the primer should have 2, 3, 4, or 5 tandem repeat units; the length of the PCR products should range from 150 to 300 bp; the position of genes should not focus on one site; the polymorphic sites should be chosen first; different combinations of tandem repeat units should be chosen on average; the repeat base in primer should be less than four; the length of the primers should be approximately 20–23 bp; the Tm value of the primers should be ~ 60 ℃; the number of consecutive As or Ts at the 5′ or 3′ region of the primer should be less than two; and repeat sequences within primer should be forbidden. The PCR products were detected by capillary electrophoresis.

Polymorphism indexes for the SSR loci, such as the number of alleles, observed heterozygosity, expected heterozygosity and polymorphism information content, were calculated by Cervus 3.0.7^76^. We used neighbor-joining methods in NTSYS (V2.10e)^77^ software to rebuild a phylogenetic tree.

### Ethics approval and consent to participate

The 11 plant materials in this paper were collected with the permission of the local authorities. The research on plants and collection of plant material complied with institutional, national or international guidelines. We also complied with the IUCN Policy Statement on Research Involving Species at Risk of Extinction and the Convention on the Trade in Endangered Species of Wild Fauna and Flora.

## Supplementary Information

Supplementary Figures.

Supplementary Table S1.

Supplementary Table S2.

Supplementary Table S3.

Supplementary Table S4.

Supplementary Table S5.

Supplementary Table S6.

Supplementary Table S7.

Supplementary Table S8.

Supplementary Table S9.

Supplementary Table S10.

Supplementary Table S11.

Supplementary Table S12.

## Data Availability

All sequencing data were deposited on NCBI (https://www.ncbi.nlm.nih.gov/) at BioProject ID: PRJNA718952.

## References

[1] Bartish IV, Garkava LP, Rumpunen K (2000). Phylogenetic relationships and differentiation among and within populations of *Chaenomeles* Lindl. (Rosaceae) estimated with RAPDs and isozymes. Theor. Appl. Genet..

[2] Song-jie, Y. Research advances on plant germplasm resources of *chaenomeles*. *Hubei Agric. Sci.* **20** (2011).

[3] Strek M, Gorlach S, Podsedek A, Sosnowska D, Hrabec E (2007). Procyanidin oligomers from Japanese Quince (*Chaenomeles japonica*) fruit inhibit activity of MMP-2 and MMP-9 metalloproteinases. J. Agric. Food Chem..

[4] Chen RL, Wu TJ, Dai YJ (2000). Studies on the chemical constiuents of four species of *Chaenomeles*. West China J. Pharm. Sci..

[5] Koehne, E. Gattungen der Pomaceen. (1890).

[6] Morley BD (1979). Augustine henry: His botanical activities in China, 1882–1890. Glasra..

[7] Galan R, Palmer J (2000). The occurrence of the rare Ciboria aestivalis in Europe. Czech Mycol..

[8] Potter D, Eriksson T, Evans RC (2007). Phylogeny and classification of Rosaceae. Plant Syst. Evol..

[9] Phipps JB, Robertson KR, Smith PG, Rohrer JR (1990). A checklist of the subfamily Maloideae (Rosaceae). Can. J. Bot..

[10] Robertson KR, Phipps JB, Smith R (1991). A synopsis of genera in Maloideae (Rosaceae). Syst Bot..

[11] Rumpunen, K., Bartish, I., Garkavagustavsson, L. & Nybom, H. Molecular and morphological diversity in the plant genus *Chaenomeles*. (2003).

[12] da Silva JAT (2018). Santalum molecular biology: Molecular markers for genetic diversity, phylogenetics and taxonomy, and genetic transformation. Agrofor. Syst..

[13] Chrungoo N (2018). Establishing taxonomic identity and selecting genetically diverse populations for conservation of threatened plants using molecular markers. Curr. Sci..

[14] Sharma, V. & Salwal, R. Molecular markers and their use in taxonomic characterization of *Trichoderma* spp. *Mol. Mark. Mycol.* 37–52 (2017).

[15] Bartish IV, Rumpunen K, Nybom H (2010). Combined analyses of RAPDs, cpDNA and morphology demonstrate spontaneous hybridization in the plant genus *Chaenomeles*. Heredity.

[16] He J (2014). Genetic variability of cultivated *Chaenomeles speciosa* (Sweet) Nakai based on AFLP analysis. Biochem. Syst. Ecol..

[17] Zhang YY (2016). Analysis of genetic diversity in *Chaenomeles* using apple EST-SSRs. Biotechnol. Bull..

[18] Julio E (2020). RNA-Seq analysis of *Orobanche* resistance in *Nicotiana tabacum*: Development of molecular markers for breeding recessive tolerance from ‘Wika’ tobacco variety. Euphytica.

[19] Thakur O, Randhawa GS (2018). Identification and characterization of SSR, SNP and InDel molecular markers from RNA-Seq data of guar (*Cyamopsis tetragonoloba*, L. Taub.) roots. BMC Genom..

[20] Wu N (2018). RNA-seq facilitates development of chromosome-specific markers and transfer of rye chromatin to wheat. Mol. Breed..

[21] Li H, Ruan CJ, Wang L, Ding J, Tian XJ (2017). Development of RNA-Seq SSR markers and application to genetic relationship analysis among sea buckthorn germplasm. J. Am. Soc. Hortic. Sci..

[22] Chen, H. Analysis of genetic diversity and relationship among chaenomeles germplasm using RAPD and AFLP markers. Master degree dissertation, Shandong Agricultural University, Tai’an. (2008).

[23] Arnold, J. & Zhuge, R. Flora of China. (2007).

[24] Carbone I, Ramirez-Prado JH, Jakobek JL, Horn BW (2007). Gene duplication, modularity and adaptation in the evolution of the aflatoxin gene cluster. BMC Evol. Biol..

[25] Hurst LD (2002). The Ka/Ks ratio: diagnosing the form of sequence evolution. Trends Genet..

[26] Carbone, I., Jakobek, J. L., RAMIREZ‐PRADO, J. H. & Horn, B. W. Recombination, balancing selection and adaptive evolution in the aflatoxin gene cluster of *Aspergillus parasiticus*. *Mol. Ecol.* **16** (2007). 10.1111/j.1365-294X.2007.03464.x 17725568

[27] Fukazawa J (2014). DELLAs function as coactivators of GAI-ASSOCIATED FACTOR1 in regulation of gibberellin homeostasis and signaling in *Arabidopsis*. Plant Cell.

[28] Fujikura U, Horiguchi G, Ponce MR, Micol JL, Tsukaya H (2010). Coordination of cell proliferation and cell expansion mediated by ribosome-related processes in the leaves of *Arabidopsis thaliana*. Plant J..

[29] Bertini L (2012). Modular structure of HEL protein from *Arabidopsis* reveals new potential functions for PR-4 proteins. Biol. Chem..

[30] Geraldo N, Bäurle I, Kidou S-I, Hu X, Dean C (2009). FRIGIDA delays flowering in *Arabidopsis* via a cotranscriptional mechanism involving direct interaction with the nuclear cap-binding complex. Plant Physiol..

[31] Faigon-Soverna A (2006). A constitutive shade-avoidance mutant implicates TIR-NBS-LRR proteins in *Arabidopsis* photomorphogenic development. Plant Cell.

[32] Kranz HD (2010). Towards functional characterisation of the members of the R2R3-MYB gene family from *Arabidopsis thaliana*. Plant J..

[33] Moreno, A. A. *et al* IRE1/bZIP60-mediated unfolded protein response plays distinct roles in plant immunity and abiotic stress responses. *PloS One*. **7**, e31944 (2012). 10.1371/journal.pone.0031944 PMC3281089 22359644

[34] Sharma R (2016). Genetic diversity estimates point to immediate efforts for conserving the endangered Tibetan sheep of India. Meta gene..

[35] Botstein D, White RL, Skolnick M, Davis RW (1980). Construction of a genetic linkage map in man using restriction fragment length polymorphisms. Am. J. Hum. Genet..

[36] Yu Y (2011). Genome structure of cotton revealed by a genome-wide SSR genetic map constructed from a BC 1 population between *Gossypium hirsutum* and *G. barbadense*. BMC Genom..

[37] Nie X (2016). Genome-wide SSR-based association mapping for fiber quality in nation-wide upland cotton inbreed cultivars in China. BMC Genom..

[38] Liu Q (2015). Genetic diversity and population structure of pear (*Pyrus* spp.) collections revealed by a set of core genome-wide SSR markers. Tree Genet Genomes..

[39] Khan MK (2016). Genome wide SSR high density genetic map construction from an interspecific cross of *Gossypium hirsutum* × *Gossypium tomentosum*. Front. Plant Sci..

[40] Robertson KR, Phipps JB, Smith R (1991). A synopsis of genera in maloideae (rosaceae). Syst. Bot..

[41] Lei, Z. L. C. H. Z. & Dekui, Z. Pollen morphology and cultivar Classification of the genus *Chaenomeles*. *Sci. Silvae Sin. ***5** (2008).

[42] Shao W, Jiang J (2020). The complete chloroplast genome sequences of two *Chaenomeles* species (*Chaenomeles cathayensis* and *Chaenomeles thibetica*). Mitochondrial DNA B..

[43] Eckert CG, Samis KE, Lougheed SC (2010). Genetic variation across species' geographical ranges: The central-marginal hypothesis and beyond. Mol. Ecol..

[44] Bartish IV, Rumpunen K, Nybom H (1999). Genetic diversity in *Chaenomeles* (Rosaceae) revealed by RAPD analysis. Plant Syst. Evol..

[45] Duarte JM (2010). Identification of shared single copy nuclear genes in *Arabidopsis*, *Populus*, *Vitis* and *Oryza* and their phylogenetic utility across various taxonomic levels. BMC Evol. Biol..

[46] Feau, N., Decourcelle, T., Husson, C., Desprez-Loustau, M. L. & Dutech, C. Finding single copy genes out of sequenced genomes for multilocus phylogenetics in non-model fungi. *PLoS One* **6** (2011). 10.1371/journal.pone.0018803 PMC3076447 21533204

[47] Li Z (2017). Single-copy genes as molecular markers for phylogenomic studies in seed plants. Genome Biol. Evol..

[48] Teasdale LC, Köhler F, Murray KD, O'hara T, Moussalli A (2016). Identification and qualification of 500 nuclear, single-copy, orthologous genes for the *Eupulmonata* (Gastropoda) using transcriptome sequencing and exon capture. Mol. Ecol. Resour..

[49] Wu F, Mueller LA, Crouzillat D, Pétiard V, Tanksley SD (2006). Combining bioinformatics and phylogenetics to identify large sets of single-copy orthologous genes (COSII) for comparative, evolutionary and systematic studies: A test case in the euasterid plant clade. Genetics.

[50] Cabrera A (2009). Development and bin mapping of a Rosaceae Conserved Ortholog Set (COS) of markers. BMC Genom..

[51] Fan X (2009). Phylogeny and evolutionary history of *Leymus* (Triticeae; Poaceae) based on a single-copy nuclear gene encoding plastid acetyl-CoA carboxylase. BMC Evol. Biol..

[52] Salas-Leiva DE (2013). Phylogeny of the cycads based on multiple single-copy nuclear genes: Congruence of concatenated parsimony, likelihood and species tree inference methods. Ann. Bot..

[53] Han F, Peng Y, Xu L, Xiao P (2014). Identification, characterization, and utilization of single copy genes in 29 angiosperm genomes. BMC Genom..

[54] Wang, J. S., Jun-Hu, H. E., Chen, H. R., Chen, Y. Y. & University, P. Comparison on the detection efficiency of different types of molecular markers in *Pineapple*. *Hubei Agricultural Sciences* (2015).

[55] Owczarek K (2017). Flavanols from Japanese quince (*Chaenomeles japonica*) fruit suppress expression of cyclooxygenase-2, metalloproteinase-9, and nuclear factor-kappaB in human colon cancer cells. Acta Biochim. Pol..

[56] Zhang, M., Mo, H., Sun, W., Guo, Y. & Li, J. Systematic isolation and characterization of cadmium tolerant genes in tobacco: A cDNA library construction and screening approach. *PLoS One*. **11**, e0161147 (2016). 10.1371/journal.pone.0161147 PMC5007098 27579677

[57] Chen S, Zhou Y, Chen Y, Gu J (2018). fastp: An ultra-fast all-in-one FASTQ preprocessor. Bioinformatics.

[58] Grabherr MG (2011). Full-length transcriptome assembly from RNA-Seq data without a reference genome. Nat. Biotechnol..

[59] Li B, Dewey CN (2011). RSEM: Accurate transcript quantification from RNA-Seq data with or without a reference genome. BMC Bioinf..

[60] Kent WJ (2002). BLAT—The BLAST-like alignment tool. Genome Res..

[61] Conesa A (2005). Blast2GO: A universal tool for annotation, visualization and analysis in functional genomics research. Bioinformatics.

[62] Li H (2020). MicroRNA comparison between poplar and larch provides insight into the different mechanism of wood formation. Plant Cell Rep..

[63] Buchfink B, Xie C, Huson DH (2015). Fast and sensitive protein alignment using DIAMOND. Nat. Methods..

[64] Li L, Stoeckert CJ, Roos DS (2003). OrthoMCL: Identification of ortholog groups for eukaryotic genomes. Genome Res..

[65] Tamura K (2012). Estimating divergence times in large molecular phylogenies. Proc. Natl. Acad. Sci. USA.

[66] Tamura K, Tao Q, Kumar S (2018). Theoretical foundation of the RelTime method for estimating divergence times from variable evolutionary rates. Mol. Biol. Evol..

[67] Retief JD (2000). Phylogenetic analysis using PHYLIP. Bioinformat. Methods Protocols.

[68] Kumar S, Stecher G, Li M, Knyaz C, Tamura K (2018). MEGA X: Molecular evolutionary genetics analysis across computing platforms. Mol. Biol. Evol..

[69] Goodman M (2009). Phylogenomic analyses reveal convergent patterns of adaptive evolution in elephant and human ancestries. Proc. Natl. Acad. Sci. USA.

[70] Wang D, Zhang Y, Zhang Z, Zhu J, Yu J (2010). KaKs_Calculator 2.0: a toolkit incorporating gamma-series methods and sliding window strategies. Genomics Proteomics Bioinform..

[71] Team, A. C. Adobe illustrator CS5 classroom in a book: ADOBE ILLUST CS5 CLASSROOM_p1. (Pearson Education, 2010).

[72] Chen, C., Chen, H., He, Y. & Xia, R. TBtools, a toolkit for biologists integrating various biological data handling tools with a user-friendly interface. *BioRxiv*. 289660 (2018).

[73] Beier S, Thiel T, Münch T, Scholz U, Mascher M (2017). MISA-web: A web server for microsatellite prediction. Bioinformatics.

[74] Andreas U, Ioana C, Triinu K, Jian Y, Faircloth BC, Maido R (2012). Primer3—New capabilities and interfaces. Nucleic Acids Res..

[75] Bartish I, Garkava L, Rumpunen K, Nybom H (2000). Phylogenetic relationships and differentiation among and within populations of *Chaenomeles* Lindl (Rosaceae) estimated with RAPDs and isozymes. Theor. Appl. Genet..

[76] Slate J, Marshall T, Pemberton J (2000). A retrospective assessment of the accuracy of the paternity inference program CERVUS. Mol. Ecol..

[77] Rohlf, F. Numerical taxonomy and multivariate analysis system version 2.1. User Guide. *Exeter Software, New York* (2000).

