# Supplementary material for: Comparative transcriptomics provides a strategy for phylogenetic analysis and SSR marker development in Chaenomeles
Source: Sci Rep. 2021 Aug 12;11:16441. doi: 10.1038/s41598-021-95776-z (PMC8361139; doi:10.1038/s41598-021-95776-z)
Supplement: Supplementary file 1 — Supplementary Figures. [file 41598_2021_95776_MOESM1_ESM.docx]

**Supplementary Information**

**Comparative transcriptomics provides a strategy for phylogenetic analysis and SSR marker development in *Chaenomeles***

Wenhao Shao^1^, Shiqing Huang^2^, Yongzhi Zhang^2^, Jingmin Jiang^1^ & Hui Li^3*^

1. Research Institute of Subtropical Forestry, Chinese Academy of Forestry, Hangzhou 311400, China.
2. Longshan Forest Farm of Anji County, Huzhou 313300, China.
3. Guangzhou Institute of Forestry and Landscape Architecture, Guangzhou, 510405, China;

*Correspondence: huil1984@163.com


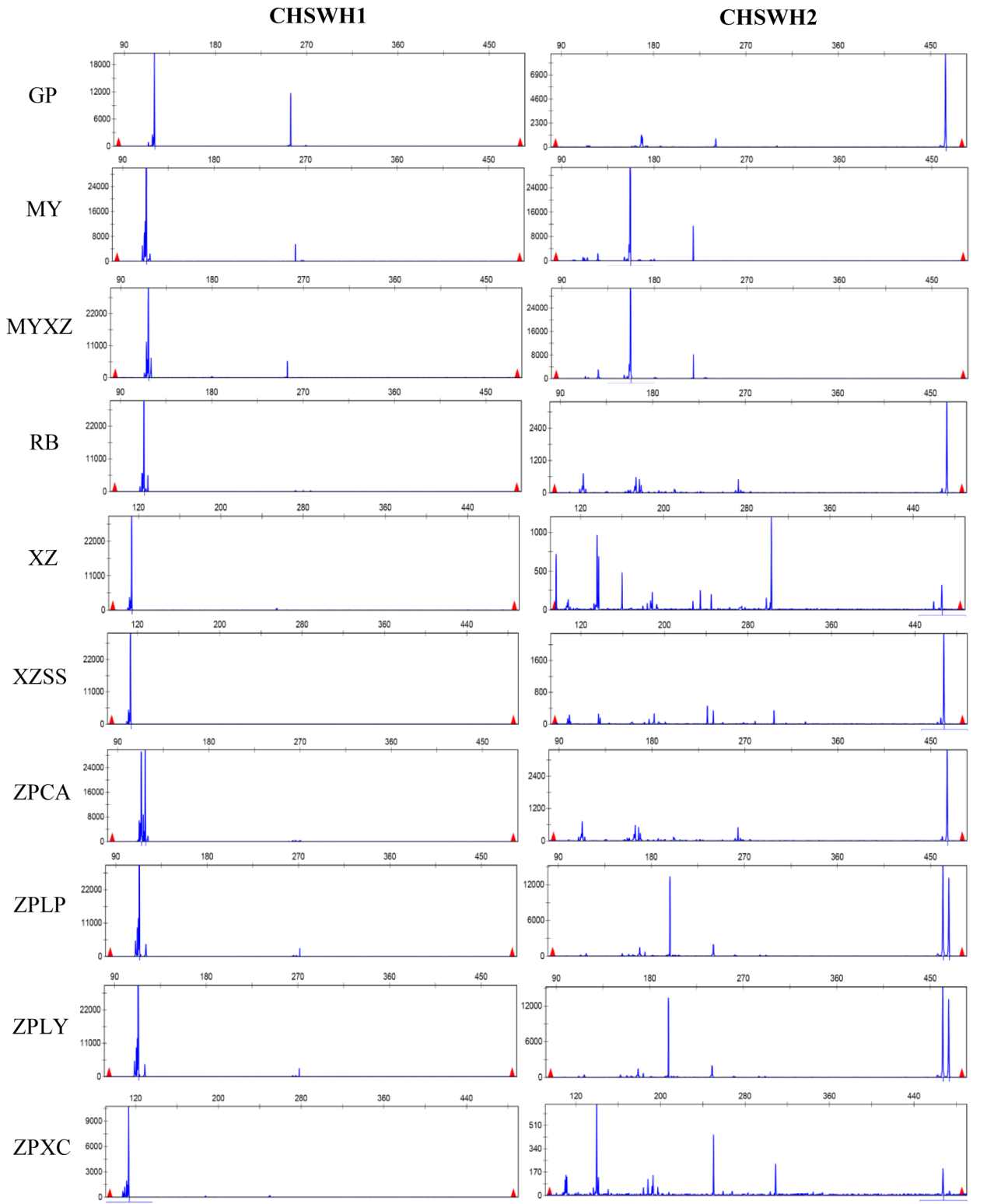


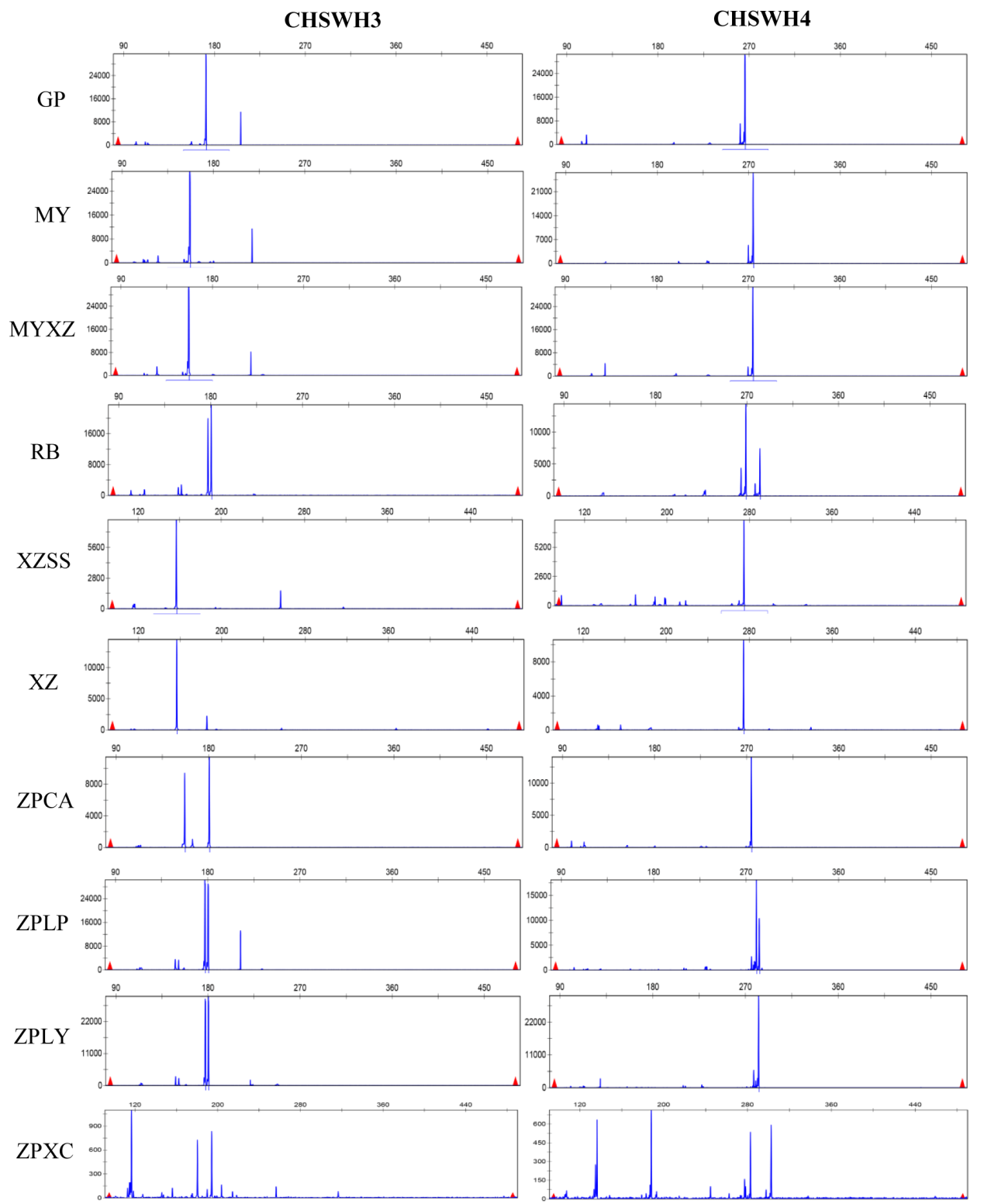


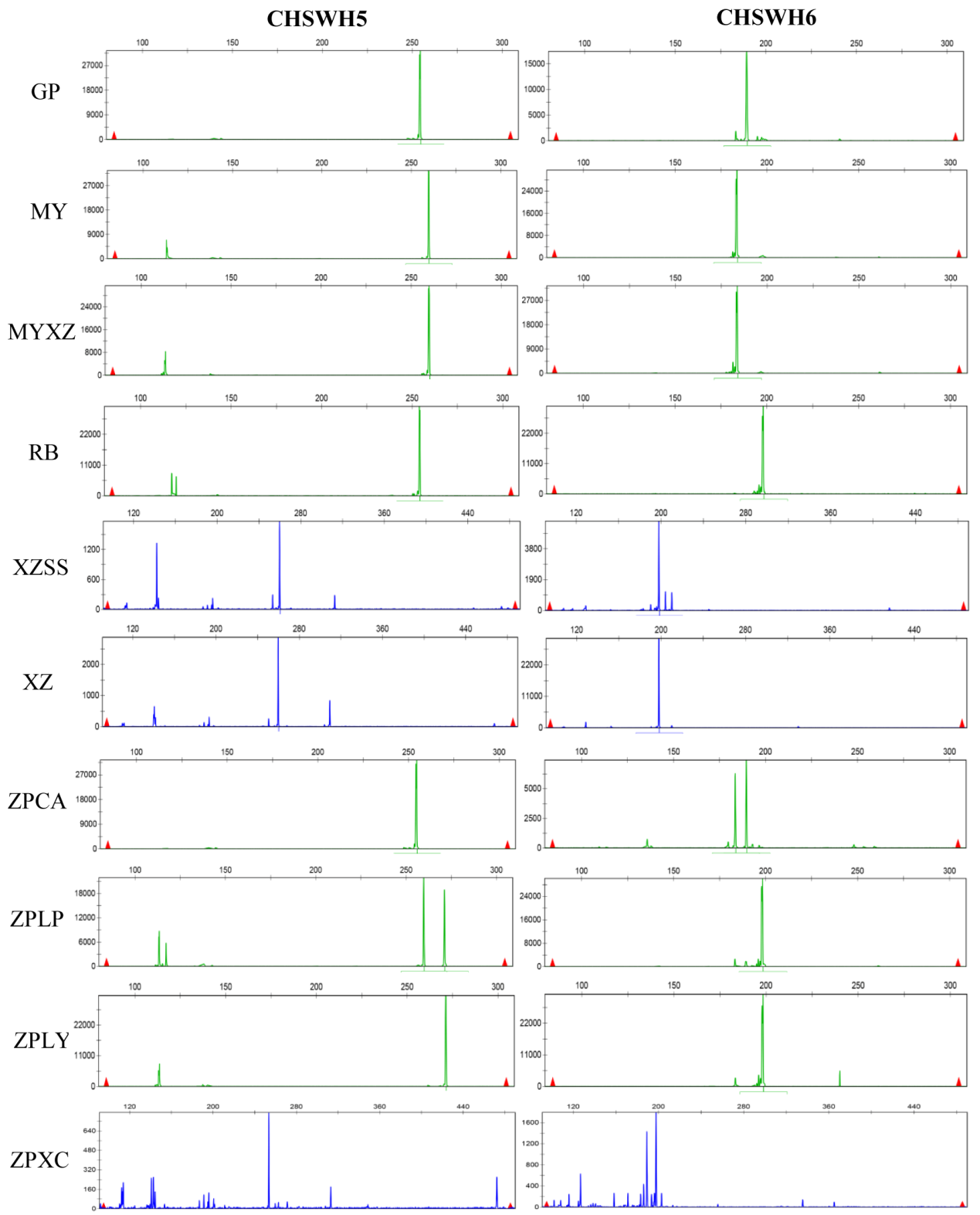


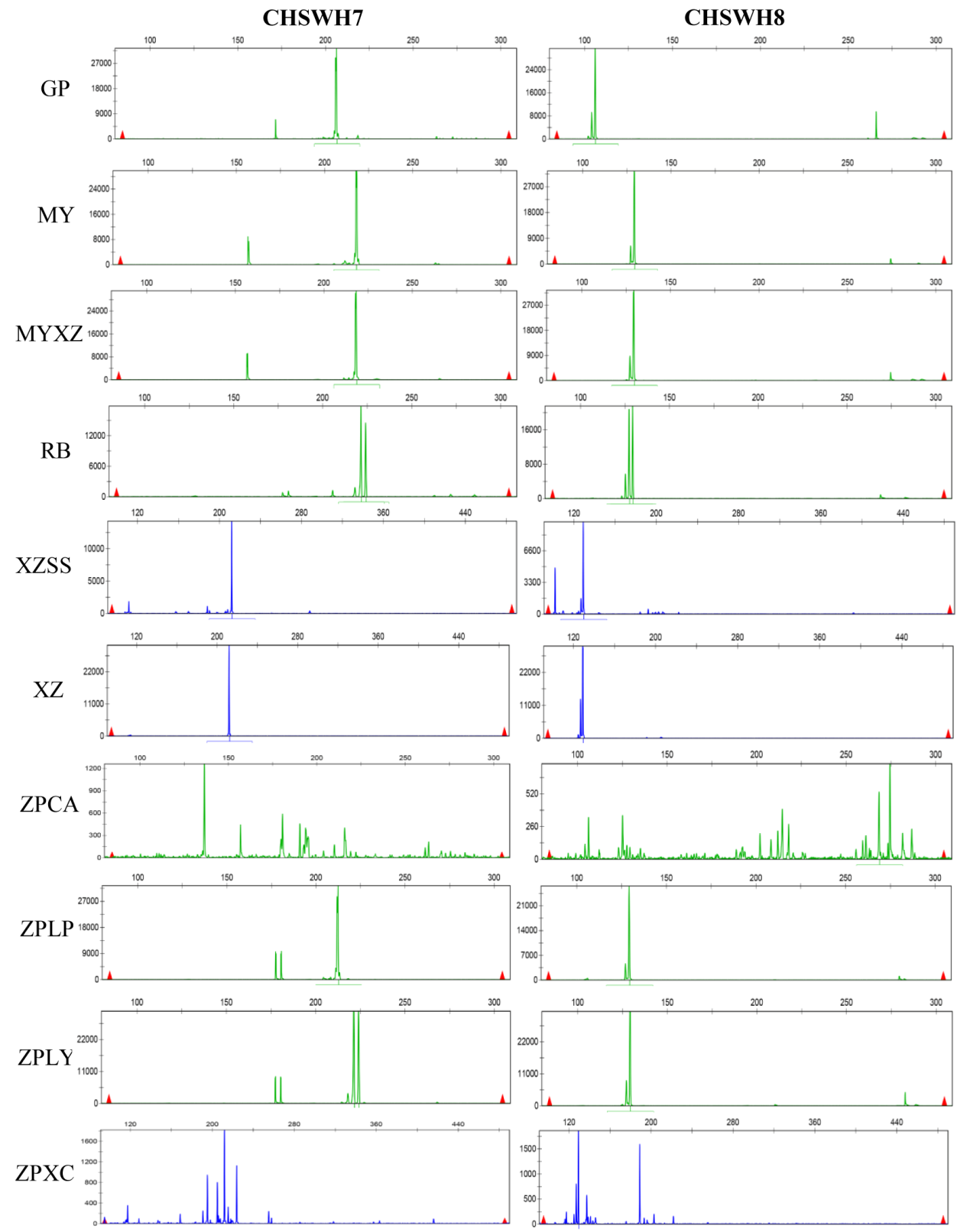


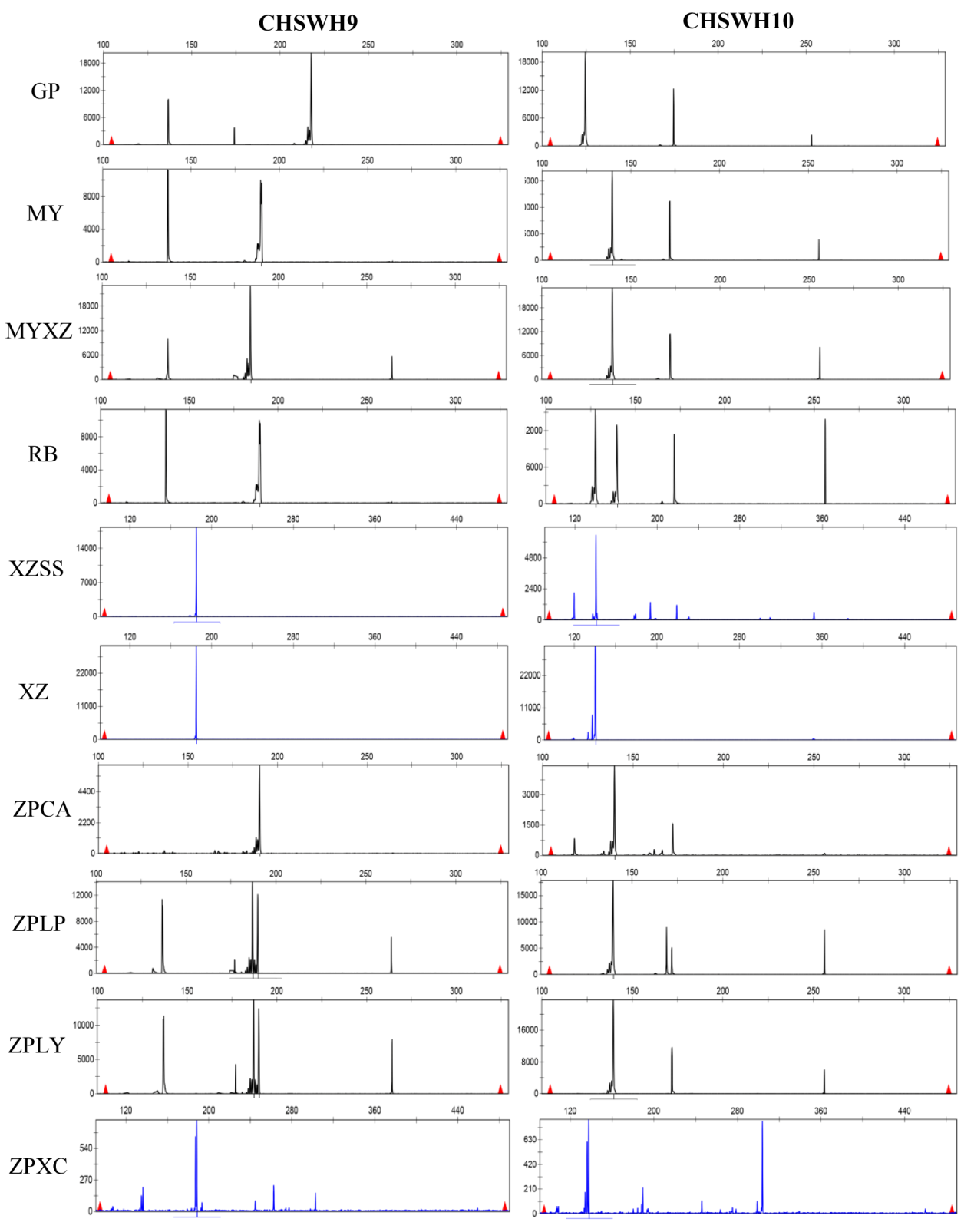


**Figure S1. Capillary electrophoresis result of 11 SSR primers.** In this graph, the x-axis represents the size of the fragment, and the y-axis represents the expression of the corresponding fragments.


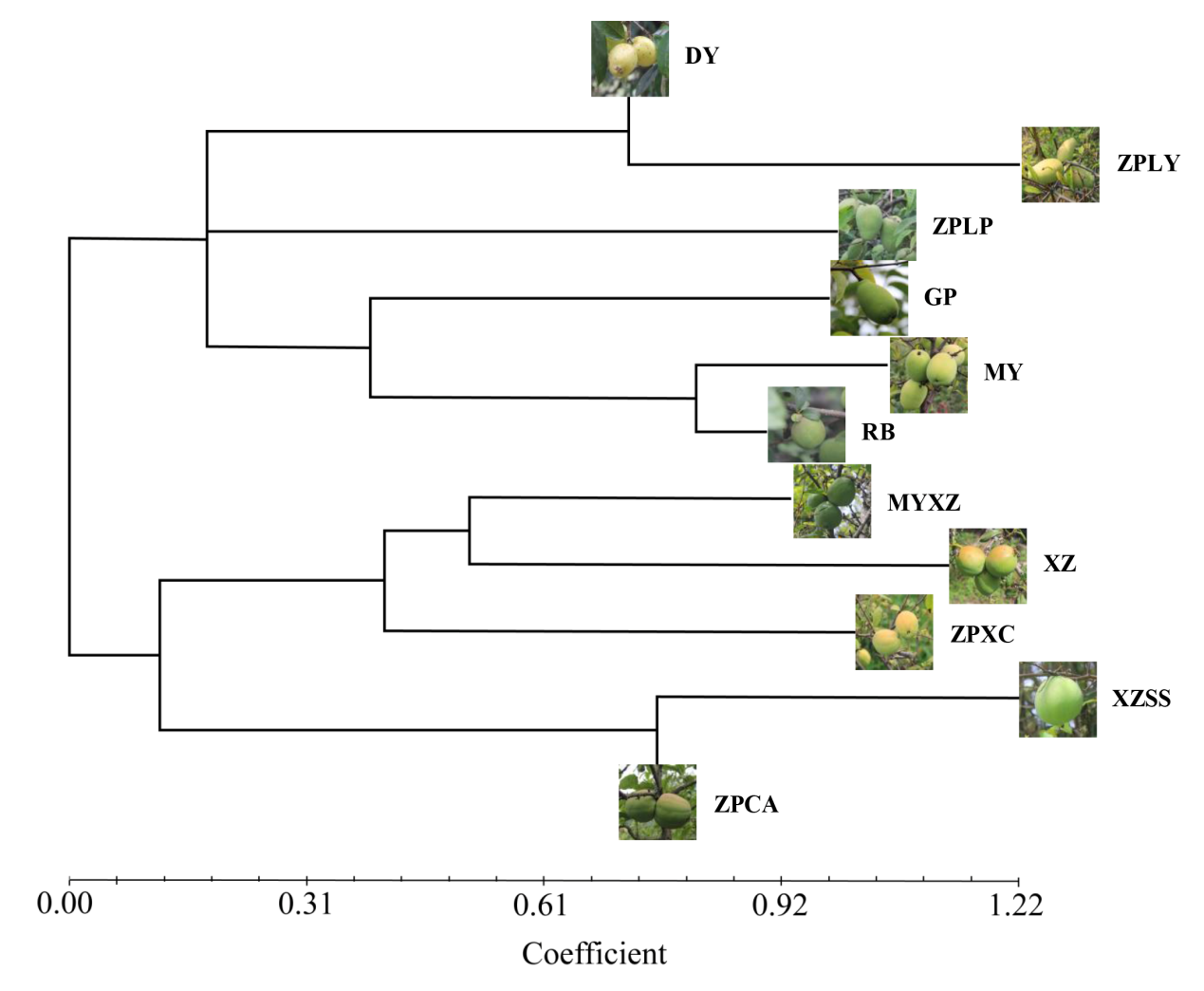


**Figure S2. Phylogenetic tree based on screened SSRs markers.** The Lateral axis in this graph indicates the heredity distance among different germplasms. The phylogenic tree was constructed by NTSYS (V 2.10e) software.
